# GDF15 Is an Eribulin Response Biomarker also Required for Survival of DTP Breast Cancer Cells

**DOI:** 10.3390/cancers14102562

**Published:** 2022-05-23

**Authors:** Chiara Bellio, Marta Emperador, Pol Castellano, Albert Gris-Oliver, Francesc Canals, Alex Sánchez-Pla, Esther Zamora, Joaquín Arribas, Cristina Saura, Violeta Serra, Josep Tabernero, Bruce A. Littlefield, Josep Villanueva

**Affiliations:** 1 Vall d’Hebron Institute of Oncology (VHIO), 08035 Barcelona, Spain; cbellio@vhio.net (C.B.); marta_emm@hotmail.com (M.E.); polcaes@gmail.com (P.C.); algrol@hotmail.com (A.G.-O.); fcanals@vhio.net (F.C.); ezamora@vhio.net (E.Z.); jarribas@vhio.net (J.A.); csaura@vhio.net (C.S.); vserra@vhio.net (V.S.); jtabernero@vhio.net (J.T.); 2 Genetics Microbiology and Statistics Department, Universitat de Barcelona, 08035 Barcelona, Spain; asanchez@ub.edu; 3 Statistics and Bioinformatics Unit, Vall d’Hebron Research Institute (VHIR), 08035 Barcelona, Spain; 4 Breast Cancer Program, Vall d’Hebron University Hospital, 08035 Barcelona, Spain; 5 Cancer Research Program, Hospital del Mar Medical Research Institute (IMIM), 08003 Barcelona, Spain; 6 Centro de Investigación Biomédica en Red de Cáncer, Monforte de Lemos, 28029 Madrid, Spain; 7 Department of Biochemistry and Molecular Biology, Universitat Autónoma de Barcelona, Campus de la UAB, 08193 Bellaterra, Spain; 8 Institució Catalana de Recerca i Estudis Avançats (ICREA), 08010 Barcelona, Spain; 9 Eisai Inc., Cambridge, MA 02140, USA; bruce_littlefield@eisai.com

**Keywords:** drug tolerance, drug tolerant persister (DTP), eribulin, growth differentiation factor 15 (GDF15), breast cancer (BC), secretome

## Abstract

**Simple Summary:**

Drug tolerant persister (DTP) cells are a unique, small sub-population of cancer cells that maintain viability under anti-cancer cytotoxic treatments. These cells enter into a reversible drug-tolerant state, which is believed to be the root of tumor recurrence. Therefore, there is a great need to find novel ways to monitor and eliminate DTP cells. We have identified the secretion of GDF15 as a response biomarker of eribulin treatment, as well as a specific biomarker of DTP cells in breast cancer. GDF15 expression is low or absent in cells sensitive to eribulin, strongly upregulated during response to the drug, and then downregulated when stable resistance is ultimately established. We have also shown that GDF15 plays a direct role in the survival of DTP cells. Thus, targeting GDF15 could help eradicate DTP cells and block the onset of stable acquired resistance. Most importantly, our data suggest that the combination of eribulin plus a GDF15 neutralizing antibody might be beneficial in the treatment of breast cancer.

**Abstract:**

Drug tolerant persister (DTP) cells enter into a reversible slow-cycling state after drug treatment. We performed proteomic characterization of the breast cancer (BC) DTP cell secretome after eribulin treatment. We showed that the growth differentiation factor 15 (GDF15) is a protein significantly over-secreted upon eribulin treatment. The biomarker potential of GDF15 was confirmed in 3D-cell culture models using BC cells lines and PDXs, as well as in a TNBC in vivo model. We also found that GDF15 is required for survival of DTP cells. Direct participation of GDF15 and its receptor GFRAL in eribulin-induction of DTPs was established by the enhanced cell killing of DTPs by eribulin seen under GDF15 and GFRAL loss of function assays. Finally, we showed that combination therapy of eribulin plus an anti-GDF15 antibody kills BC-DTP cells. Our results suggest that targeting GDF15 may help eradicate DTP cells and block the onset of acquired resistance.

## 1. Introduction

Overcoming acquired resistance is one of the greatest challenges in the management of cancer patients. Although patients often have an initial positive response to cancer drugs, many will eventually stop responding to treatment. Through the years, different views on how tumor cells acquire drug resistance have been adopted. Since tumorigenesis is largely driven by genetic mutations, drug resistance was initially proposed as being driven by the acquisition of mutations that allow tumor cells to overcome the drugs’ actions [1]. This is indeed true in many cases, for example in the acquisition/enrichment of KRAS mutations in colorectal cancer during treatment with cetuximab [2]; or with de novo mutations occurring in EGFR during treatment with tyrosine kinase inhibitors in lung cancer [3]. In recent years, however, several examples have been described of drug resistance mediated through non-genetic mechanisms [4,5,6,7]. In general, non-genetic drug acquired resistance involves major transcriptional changes driven by epigenetic reprogramming in a very small fraction of tumor cells [8,9]. A more recent emerging concept with the potential to change the drug resistance framework is drug tolerance [10]. Originally observed during the treatment of bacteria with antibiotics [11], drug tolerance refers to a transitory state in which a small percentage of cells can survive high concentrations of a drug by entering into a so-called slow-cycling phase; such cells are often referred to as drug-tolerant persister (DTP) cells [12,13].

The concepts of drug tolerance and DTP have intriguing implications for biomarker discovery since they both tolerate (survive) the drug treatment, yet also reflect the initial drug response in a pharmacodynamic sense. Thus, DTPs represent both positive (initial response) and negative (transition toward full resistance) therapeutic aspects, i.e., the Yin and Yang of response to drug treatment.

Eribulin is a fully synthetic macrocyclic ketone analog of the structurally complex marine sponge natural product halichondrin B [14]. As a microtubule targeting agent (MTA), eribulin (in its clinical formulation of eribulin mesylate) has gained regulatory approval in many countries worldwide for the treatment of certain patients with advanced breast cancer, liposarcoma, or soft tissue sarcoma (according to country-specific approval indications). Eribulin acts by binding to terminal β-tubulin subunits at the growing plus (+) ends of microtubules, thus inhibiting the growth phase of microtubule dynamics while having no direct effect on microtubule shortening, a mode of action distinct from other MTAs which likely contributes to its clinical efficacy and tolerability profile [15,16,17,18,19]. Moreover, preclinical studies indicate that eribulin also exerts effects unrelated to its anti-mitotic effects, including vascular remodeling, reversal of the epithelial-to-mesenchymal (EMT) transition, inhibition of cancer cell migration/invasion and alterations in cellular phenotype, with growing clinical evidence supporting the concept that these non-cytotoxic effects likely contribute to eribulin’s clinical efficacy [20,21]. However, the complex interplay of both cytotoxic and non-cytotoxic effects may be different in different patients, therefore the need for the development of non-invasive circulating biomarkers to both monitor the response to therapy as well as identify patients who either do not respond to eribulin or are headed toward the development of primary resistance.

Since eribulin disrupts the cytoskeleton, and hence intracellular vesicle trafficking [22], we hypothesized that eribulin treatment would alter the secretome of breast cancer cells. Thus, in this study, we sought to employ quantitative proteomics to characterize the secretome of breast cancer cells following eribulin treatment. Here, we show that eribulin induces a population of metastable DTP cells which are characterized by a specific secretome. Among the many proteins significantly over-secreted upon eribulin treatment, growth differentiation factor 15 (GDF15; originally called macrophage inhibitory cytokine-1 or MIC-1), seemed particularly promising and was selected for further validation. GDF15 is a cell stress cytokine belonging to the TGF-β superfamily. Different cell stressors and pathological conditions trigger increased serum levels of GDF15 [23]. Elevated levels of circulating GDF15 cause anorexia and weight loss through binding to its receptor GDNF family receptor alpha-like (GFRAL), which in turn enables signaling through the proto-oncogene tyrosine kinase RET [24,25,26]. In cancer, GDF15 mediates cachexia, causing extreme weight loss and muscle wastage in late-stage cancer patients [27,28,29]. Intriguingly, in our studies, GDF15 was only detected during the metastable DTP state following eribulin treatment, yet disappears after the acquisition of full drug resistance following prolonged eribulin treatment. Results obtained in cell lines grown in two-dimensional culture were further confirmed using breast cancer three-dimensional organoid derived from both established cell lines and PDX models. Finally, we tested and confirmed the hypothesis that GDF15 itself plays a role in maintaining the DTP state by conducting GDF15 loss of function experiments, results of which led to a proof-of-concept study combining eribulin with an anti-GDF15 antibody to specifically inhibit the generation of DTP cells in both TNBC and luminal breast cancer cell lines.

## 2. Material and Methods

### 2.1. Cell Culture and Breast Cancer PDX Tumor Cells Isolation

Breast cancer cell lines MDA-MB-231, MCF7, HS578T, BT549, HCC1937 and MCF10A were purchased from the American Type Culture Collection (ATCC). The cells were cultured in 5% CO_2_ and 95% humidified atmosphere air at 37 °C. The cell lines were authenticated by short tandem repeat profiling (IdentiCell, Aarthus University Hospital). All cell lines were routinely tested by PCR for *Mycoplasma* and passaged until passage number 15. MDA-MB-231, MCF7 and HS578T cells were maintained in DMEM: Nutrient Mixture F12 (DMEM/F12; Invitrogen, Waltham, MA, USA) supplemented with 10% FBS (Invitrogen), 1% Pen/Strep (Thermo Fisher Scientific, Waltham, MA, USA) and 2 mmol/L l-Glutamine (Invitrogen). BT549 and HCC1937 cells were maintained in RPMI (Invitrogen) supplemented with 10% FBS (Invitrogen), 1% Pen/Strep (Thermo Fisher Scientific) and 2 mmol/L l-Glutamine (Invitrogen). MCF10A cells were maintained in DMEM: Nutrient Mixture F12 (DMEM/F12; Invitrogen) supplemented with 10% FBS (Invitrogen), 1% Pen/Strep (Thermo Fisher Scientific), 2 mmol/L l-Glutamine (Invitrogen), 20 ng/mL human EGF (#AF-100-15, Peprotech, Cranbury, NJ, USA), 10 μg/mL insulin (#I9278, Sigma-Aldrich, St. Louis, MO, USA) and 500 ng/mL hydrocortisone (#H0888, Sigma-Aldrich). Breast cancer PDXs were kindly provided by the Experimental Therapeutics Group headed by Violeta Serra at the VHIO institute. Breast cancer samples were taken from resected tumors and underwent multiple washes with PBS before minced into small pieces using a scalpel and incubated with human collagenase (3 mg/mL, Sigma) and hyaluronidase (1 mg/mL, Sigma) for 1 h at 37 °C with shacking at 200–300 rpm. After incubation, the mixture was resuspended in DMEM-HEPES 1% and centrifuged at 1500 rpm for 5 min to remove debris and residual collagenase and hyaluronidase. After the wash, the cell pellet was resuspended in DMEM-HEPES 1% and filtered through a 40 μm cell strainer to remove large undigested fragments. The cell suspension was centrifuged at 200× *g* for 3 min. The cell pellet was resuspended in Trypsin-EDTA (Sigma) and gently pipetted up and down with a p1000 pipette for 3 min at RT. The effect of the Trypsin was blocked adding cold Hank’ Balanced Salt Solution (Biowest, Riverside, MO, USA) supplemented with 2% FBS and 2% HEPES and the cell suspension was centrifuged at 1500 rpm for 5 min. After removal of the supernatant, pre-warmed Dispase (5 mg/mL; Sigma) and DNase I (1 mg/mL; Sigma) were added. The samples were pipetted for 3 min with a p1000 pipette for further dissociation of cell clumps. After washing in cold HF-2% HEPES 2% FBS, cell pellets were resuspended in RBC lysis buffer (eBioscience, San Diego, CA, USA) and incubated for 3 min at RT to lysis erythrocytes. After a final washing in cold HF-2% HEPES 2% FBS, cells were resuspended in DMEM/F12 medium supplemented with 10% FBS and counted. The isolated breast cancer PDX cells were resuspended at the concentration of 50,000 cells/drop of Matrigel matrix and plated. Detailed protocol for breast cancer organoids is provided in the specific session. Cellular images were taken at different enlargements with the microscope Leica MCF170 HD.

### 2.2. Three-Dimensional Cell Culture Organoids

Cell suspension (from breast cancer cell lines or PDXs derived cells) was resuspended in serum-free DMEM/F12 media to wash the cells from FBS that could interfere in organoid formation and centrifuged at 1500 rpm for 5 min. Cells were resuspended in serum-free DMEM/F12 media, counted and divided into 50,000 cells/Eppendorf for each condition. Cells were resuspended in a solution 60% BD-Matrigel matrix (Cultek S.L.U.) and 40% growth medium-organoid medium consisting of DMEM/F12, B27 Supplement (1:50; Gibco), and insulin (250 ng/mL; Life Technologies)- and plated as 50 μL droplets in a 24-well plate. The Matrigel drops were incubated for 30 min at 37 °C and then organoid media was carefully added to each well. Media with fresh growth factors was changed every 3 days.

### 2.3. Drug Treatment (Eribulin, Vinorelbine and Paclitaxel)—CellTiter, Cell Counting and Crystal Violet Staining

Eribulin was provided by Eisai Inc. Vinorelbine and Paclitaxel was provided by the Pharmaceutical Centre of Vall d’Hebron Hospital. IC_50_ values (the concentration required to kill and/or inhibit growth of cells by 50% as compared with untreated control wells) of these drugs were estimated from concentration–response curves by using CellTiter colorimetric assay (CellTiter-Blue Cell Viability Assay; Promega) analysis. All the cell lines were plated in 96-well plates (5000 cells/well in 100 μL media). After overnight incubation, Eribulin (0.1, 0.5, 1, 2 nmol/L), Vinorelbine (5, 10, 50, 100 nmol/L), and Paclitaxel (1, 2, 4, 10 nmol/L) were added in six replicates to each population. Cell viability was measured after 7 days using the CellTiter assay. A calibration curve was prepared using the data obtained from wells that contained a known number of cells. Cell proliferation was assessed by Trypan Blue (Thermo Fisher Scientific, Waltham, MA, USA) staining and cell counting following treatment with the indicated concentrations of each drug. Crystal violet staining was performed to collect representative images of drug treatment effect on cell viability. Specifically, vehicle and drug-treated cells were washed twice with cold PBS and fixed for 10 min with ice-cold 100% methanol. The methanol was aspired and cells incubated with 0.5% crystal violet solution in 25% methanol for 20 min. The cells were washed several times and left to dry overnight. Crystal violet staining was quantified dissolving the dye in 10% acid acetic and measuring the absorbance at 595 nM using a luminometer. All the drug treatments were 7 days with retreatment every 72 h due to the half-lives of the drugs. 

### 2.4. Immunofluorescence and Confocal Microscopy

Cells were seeded on Collagen-coated (Collagen I-Rat Tail; Life Technologies, Carlsbad, CA, USA) glass coverslips. After growing them for the appropriate time, cells were treated according to eribulin treatment scheme and cells were fixed with paraformaldehyde 4% for 30 min and permeabilized with 0.5% Triton-X10 for 15 min. For Tubulin staining, cells were fixed in cold methanol for 10 min on dry ice. Unspecific binding sites were blocked by incubating with 3% BSA (Sigma-Aldrich) for 1 h. Phalloidin (Phalloidin–Tetramethylrhodamine B isothiocyanate; Sigma-Aldrich) was used to stain actin cytoskeleton of cells (1:1000, incubation 15 min at room temperature). Primary antibodies were prepared in 3% BSA and incubated O/N at 4 °C: mouse anti-tubulin (1:500, Sigma-Aldrich), rabbit anti-GDF15 (1:200, ThermoFisher). Primary antibody incubations were followed by PBS washes and incubation for 1 h at room temperature with the appropriate secondary antibody (goat anti-mouse and goat anti-rabbit) conjugated to Alexa Fluor 594 and Alexa Fluor 488 (Invitrogen). Nuclei were stained with Hoechst 33342 (5 μg/mL; Sigma-Aldrich). The coverslips were mounted in slides using Prolong Diamond Antifade Mountant solution (Invitrogen). 

Immunostaining of 3D-organoid cells was performed on glass coverslips where cells were grown in Matrigel matrix. Matrigel matrix was disaggregated with incubation for 20 min in ice with Corning Cell Recovery Solution (Cultek). After this incubation, the previously described protocol was followed using 1% of Triton-X10 during the permeabilization incubation. 

Immunostaining breast cancer PDX tumor samples was performed on paraffin-embedded tissues. Tissue blocks were sectioned, mounted on microscope slides, and heated at 56 °C O/N. Paraffin was removed with xylene, and tissues were serially rehydrated through descending ethanol concentrations to water. Sections were stained with hematoxylin and eosin (H&E) to assess cellular morphology. For immunofluorescence, antigen retrieval was performed by boiling the samples in EDTA (Calbiochem, San Diego, CA, USA) buffer 9941 mmol/L (pH 8), using a microwave oven. Slides were then washed twice in PBS and once in PBS-1% Tween-20 (Sigma-Aldrich) for 15 min. Unspecific binding sites, for both cells and tissues, were blocked by incubating with 3% BSA (Sigma-Aldrich) for 1 h. Primary antibodies were prepared in 3% BSA and incubated O/N at 4 °C: rabbit anti-GDF15 was used at 1:200 for tissues, and mouse anti-Cytokeratin (clones AE1/AE3; Dako GmbH, Glostrup, Denmark) was used at 1:100 for tissue samples. Primary antibody incubations were followed by PBS washes and incubation for 1 h at room temperature with the appropriate secondary antibody (goat anti-mouse and goat anti-rabbit) conjugated to Alexa Fluor 594 and Alexa Fluor 488 (Invitrogen). Nuclei were stained with Hoechst 33342 (5 mg/mL; Sigma Aldrich). 

A confocal microscope (Nikon Eclipse Ti) was used to visualize fluorescence and acquire images from five representative fields of each sample. GDF15 expression was measured in confocal images using Image J software.

### 2.5. Western Blotting

Whole-cell lysates were prepared from cell lines or 3D-organoid cells with RIPA lysis buffer supplemented with phosphatase, protease, and kinase inhibitors (all from Sigma–Aldrich). To collect cell lysates from 3D-organoid cells, the Matrigel was disaggregated as previously described (1 h of incubation). The protein concentration of the resulting supernatants was assessed using Pierce BCA protein assay kit (Thermo Fisher Scientific, Waltham, MA, USA), and ten to sixty micrograms of protein lysates were resolved on specific percentage of gel in agreement with the molecular weight of the proteins under study and transferred to polyvinylidene difluoride (PVDF) membranes (Millipore, Burlington, MA, USA). Following transference, assessment of protein loading was visualized using Ponceau Solution (Sigma–Aldrich, St. Louis, Missouri, USA), and membranes were then blocked in 5% non-fat milk in TBST. Primary antibodies were incubated overnight at 4 °C: mouse anti-p21^Cip1^ (1:1000, ThermoFisher Scientific), mouse anti-actin (1:10,000, Sigma–Aldrich), rabbit anti-EIF3L (1:1000, Proteintech), rabbit anti-EIF3B (1:1000, Proteintech), rabbit anti-KIF5B (1:1000, Proteintech), rabbit anti-GDF15 (1:1000, Proteintech), rabbit anti-GFRAL (1:1000, ThermoFisher), rabbit anti-YARS (1:1000, Life Technologies), rabbit anti-QARS (1:1000, AB Clonal), rabbit anti-DYNC1H1 (1:1000, Proteintech), rabbit anti-NUMA1 (1:1000, Novus Biologicals), rabbit anti-CSE1L (1:1000, Proteintech). Membranes were then incubated with either a mouse or rabbit horseradish peroxidase (HRP)-conjugated secondary antibody at a 1:1000 (VWR) and developed using a chemiluminescent detection reagent (ProSignal Dura, Genesee Scientific, San Diego, CA, USA). All images were captured using the BioRad ChemiDoc Imaging system and analyzed using Image J (Bethesda, MD, USA).

### 2.6. Cell Proliferation Assays: Cell Cycle and PKH26 Staining

Cell proliferation was analyzed by flow-cytometry with cell-cycle analysis and PKH26 staining analysis. For cell-cycle analysis cells were treated with either vehicle or 1.5 nM eribulin for 7 days. The cells were then fixed in 70% ethanol, incubated with 10 μg/mL RNase A (Sigma-Aldrich) at room temperature, and stained with 2 μg/mL DAPI (Hoechst 33342, Sigma-Aldrich, St. Louis, MO, USA). The DNA content of the cells (100,000 events per experimental group) was quantified using a BD-Celesta cytofluorimeter (Beckton Dickinson) and analyzed using cell-cycle software of FlowJo 10.2 version. For PKH26 staining, the PKH26 Fluorescent Cell Linker Kits (Sigma Aldrich, St. Louis, MO, USA) were used following the manufacturer’s protocol. The PKH26 staining was quantified at times 0 h, 72 h and 7 days of 1.5 nM eribulin treatment by flow cytometry (BD-Celesta cytofluorimeter; Beckton Dickinson, Franklin Lakes, NJ, USA) and collecting pictures with epifluorescence microscope (Nikon Eclipse Ti, Tokyo, Japan).

### 2.7. Secretome Collection

Two-dimensional and three-dimensional secretomes were prepared using a modified protocol of the previously described protocol developed in our laboratory [30].

*Two-dimensional secretomes*. Briefly, 4 × 10^6^ cells in exponential phase were seeded in 150 cc tissue culture plates and allowed to grow for 48 h. After that, media was aspirated, and cells were washed 5 times, 2 times with PBS and the last 3 with serum-free media. Then, cells were maintained for 8 h in the presence of serum-free media before collecting the conditioned media (secretome). 

*Three-dimensional secretome*. Three-dimensional organoids were set up as explained in the 3D cell culture organoids section. For the secretome collection, cells were collected after 8 h of incubation in serum-free media. First Matrigel matrix was disaggregated with incubation for 1 h in ice with Corning Cell Recovery Solution (Cultek). Then 3D-organoids were plated as 50 μL droplets in a 24-well plate using a solution of: Collagen I − Rat Tail (Life Technologies) + serum-free media + NaOH for titration. The 3D-organoids drops were incubated for 30 min at 37 °C and then serum-free media was carefully added to each well.

The conditioned media of both 2D and 3D cell cultures was spun down at 200 g for 5 min, and the supernatants were collected and filtered through a Millex-GP 0.22 μm pore syringe-driven filter (Millipore). Then, secretomes were concentrated using a 10,000 MWCO Millipore Amicon Ultra (Millipore). Protein concentration was determined with a Pierce BCA protein assay kit (Thermo Scientific).

Concentrated secretome samples (15 μg of total protein) were taken to 40 μL of 6 M Urea, 50 mM ammonium bicarbonate for tryptic digestion. Samples were first reduced with DTT to a final concentration of 10 mM, for 1 h at RT, and then alkylated with 20 mM of iodoacetamide for 30 min at RT in the dark. Carbamidomethylation reaction was quenched by addition of N-acetyl-L-cysteine to final concentration of 35 mM followed by incubation for 15 min at RT in the dark. Samples were diluted with 50 mM ammonium bicarbonate to a final concentration of 1M Urea, modified porcine trypsin (Promega, Madison, WI, USA) was added in a ratio of 1:10 (*w*/*w*), and the mixture was incubated overnight at 37 °C. The reaction was stopped with formic acid (FA) at a final concentration of 0.5%. The digests were then purified using SCX micro columns (PolyLC, Columbia, MD, USA). Purified samples were evaporated to dryness, dissolved in 5% acetonitrile-0.1% formic acid, and kept frozen at −20 °C until analyzed.

### 2.8. Liquid Chromatography–Mass Spectrometry Analysis (LC–MS) and Protein Identification

Tryptic digests were analyzed using a linear ion trap Velos-Orbitrap mass spectrometer (Thermo Fisher Scientific, Bremen, Germany). Instrument control was performed using Xcalibur software package, version 2.2.0 (Thermo Fisher Scientific, Bremen, Germany). Peptide mixtures were fractionated by online nanoflow liquid chromatography using an EASY-nLC 1000 system (Proxeon Biosystems, Thermo Fisher Scientific, Waltham, MA, USA) with a two-linear-column system. Samples were first loaded onto a trapping guard column (Acclaim PepMap 100 nanoviper, 2 cm long, ID 75 μm and packed with C18, 3 μm particle size from Thermo Fisher Scientific) at 4 μL/min. Then, samples were separated on the analytical column (Dr Maisch, 25 cm long, ID 75 μm, packed with Reprosil Pur C18-AQ, 3 μm particle size). Elution was performed using 0.1% formic acid in water (mobile phase A) and acetonitrile with 0.1% formic acid (mobile phase B), with a linear gradient from 0 to 35% of mobile phase B for 120 min at a flow rate of 300 nL/min. Ions were generated applying a voltage of 1.9 kV to a stainless-steel nano-bore emitter (Proxeon, Thermo Fisher Scientific), coupled to end of the analytical column, on a Proxeon nano-spray flex ion source.

The LTQ Orbitrap Velos mass spectrometer was operated in data-dependent mode. A scan cycle was initiated with a full-scan MS spectrum (from *m*/*z* 300 to 1600) acquired in the Orbitrap with a resolution of 30,000. The 20 most abundant ions were selected for collision-induced dissociation fragmentation in the linear ion trap when their intensity exceeded a minimum threshold of 1000 counts, excluding singly charged ions. Accumulation of ions for both MS and MS/MS scans was performed in the linear ion trap, and the AGC target values were set to 1 × 10^6^ ions for survey MS and 5000 ions for MS/MS experiments. The maximum ion accumulation time was 500 and 200 ms in the MS and MS/MS modes, respectively. The normalized collision energy was set to 35%, and one microscan was acquired per spectrum. Ions subjected to MS/MS with a relative mass window of 10 ppm were excluded from further sequencing for 20 s. For all precursor masses, a window of 20 ppm and isolation width of 2 Da were defined. Orbitrap measurements were performed enabling the lock mass option (*m*/*z* 445.120024) for survey scans to improve mass accuracy.

### 2.9. Protein Identification and Quantitative Differential Analysis

LC–MS/MS data were analyzed using the Proteome Discoverer v. 2.1 software (Thermo Fisher Scientific, Waltham, MA, USA). Proteins were identified using Mascot v. 2.5 (Matrix Science, London, UK) to search the SwissProt database (2018_11, taxonomy restricted to human proteins, 20,413 sequences). MS/MS spectra were searched with a precursor mass tolerance of 10 ppm, fragment tolerance of 0.7 Da, trypsin specificity with a maximum of 2 missed cleavages, cysteine carbamidomethylation set as fixed modification and methionine oxidation as variable modification. 

Files generated from Mascot (DAT files) were loaded into Scaffold (version 3.00.07; Proteome software, Inc., Portland, OR, USA), resulting in a no redundant list of identified proteins per sample. Peptide identification was given as valid as long as a PeptideProphet probability greater than 95% was determined. Those proteins whose identification could be established with a probability higher than 95% and contained at least two identified spectra were accepted. Using these filters, a false protein discovery rate (FDR) below 1.0%, as estimated by a database search, was achieved. The generated “scaffold” files containing all the “spectral counts” (SpC) for each sample and their replicates were exported to the POMAcounts 1.1.0 software for normalization and statistical analysis (see statistical analysis section).

### 2.10. siRNA Experiment

siRNA sequences directed against GDF15 and GFRAL were purchased from Dharmacon (Horizon Discovery) and used to knockdown GDF15 and GFRAL in MCF7 and MDA-MB-231 cell lines. Briefly, cells were seeded into 100 cc tissue culture plates at a density of 1.5 × 10^6^ cells/plate and the following day were treated for 72 h with 1.5 nM eribulin. After treatment, plasmid carrying, respectively, siRNA GDF15 or siRNA GFRAL and siRNA non-targeting (250 ng/plate) was transfected into the cells using RNAiMAX lipofectamine (Thermo Fisher). The media was changed after 24 h of transfection and the cells were plated according to the experimental plan to perform 7 days of 1.5 nM eribulin treatment. siGDF15 or siGFRAL cells were compared to the cells transfected with non-target siRNA plasmid.

- ON-TARGETplus Human GDF15, SMARTpool:
  UGGUUUACAUGUCGACUAA
  UGGUUUACAUGUUGUGUGA
  UGGUUUACAUGUUUUCUGA
  UGGUUUACAUGUUUUCCUA
- ON-TARGETplus Human GFRAL, SMARTpool:
  AAACAUGCUUGGAGAGUAA
  GUGAGGAAUCUUUGUGUAA
  GCAACCACGUCAAGACAAC
  CAGUUGGCCUCUUACCUUA

### 2.11. Anti-GDF15 Experiment

In vitro treatment with combination of eribulin and human antibody anti-GDF15 was performed using the commercialized rabbit anti-GDF15 from ThermoFisher (#42-1700). Briefly, cells were seeded into 96-well tissue culture plates at a density of 5000 cells/well and the following day were treated for 72 h with 1.5 nM eribulin. After treatment, eribulin (1.5 nM), and anti-GDF15 (2.5 μg/mL) were added in six replicates to each population. Cell viability was measured after 7 days using the CellTiter (Promega) assay for 2D cell-culture settings and CellTiter-Glo (Promega) for 3D cell-culture settings. Crystal violet staining was performed to collect representative images of combinational drug treatment effect on cell viability performing the experiment in 24-well plates. A rabbit IgG isotype control was used as control.

### 2.12. In Vivo Experiment

PDX tumor tissues were collected after 1 month of eribulin treatment in vivo according to the following protocol: eribulin mesylate weekly on days 1, 3, 5 intravenously (0.1 mg/kg, in PBS). Further details are described in the mentioned paper [31]. Collection of tumor samples and establishment of PDXs fresh tumor samples from patients with BC were collected following an Institutional Research Board-approved protocol and the associated written informed consent. The study was compliant with the Declaration of Helsinki. Experiments were conducted following the European Union’s animal care directive (2010/63/EU) and were approved by the Ethical Committee of Animal Experimentation of the Vall d’Hebron Research Institute, the Catalan Government or the National Research Ethics Service, Cambridgeshire ([32] and https://caldaslab.cruk.cam.ac.uk/bcape (accessed on 14 April 2022)).

### 2.13. Statistical Analysis

Data were analyzed with GraphPad Prism 6.0 (GraphPad Software 6.0, Inc.). Data from replicate experiments are shown as mean values ± standard deviation. Comparisons between groups were analyzed by a two-tailed Student *t*-test or two-way ANOVA, as appropriate. A *p*-value < 0.05 was considered statistically significant.

All statistical normalizations and calculations of proteomic data were performed using the software POMAcounts 1.1.0. Exploratory data analysis was performed using principal component analysis (PCA) and hierarchical clustering of the samples in the SpC matrix to find possible outliers and patterns in the data. For statistical modeling, a GLM model based on the Poisson distribution was used as a statistical test. Adjusted *p*-value < 0.05, fold change > 0.8 and number spectral counts (SpC) > 4 thresholds were applied during the analysis.

## 3. Results

### 3.1. Eribulin Treatment Leads to Formation of a Drug-Tolerant Persister (DTP) Subpopulation in Breast Cancer Cell Lines

**Figure 1:**
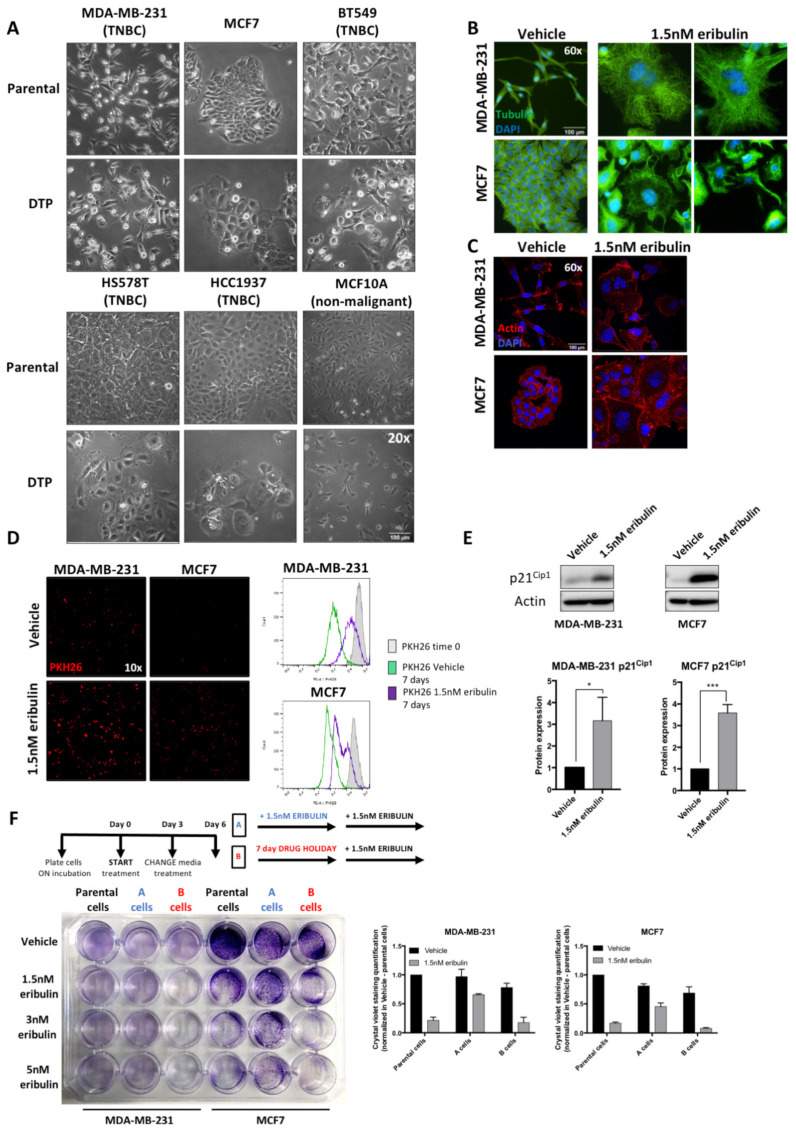
Eribulin treatment in vitro leads to the formation of a drug-tolerant persister (DTP) subpopulation. (**A**) (Upper panel) (Lower panel) Representative images of parental (cells vehicle-treated for 7 days) and DTP (cells treated for 7 days with 1.5 nM eribulin) in five different breast cancer cell lines: MDA-MB-231, MCF7, BT549, Hs579T, HCC1937. (**B**,**C**) Representative confocal images of Tubulin (green) and Actin (red) immunofluorescence in MDA-MB-231 and MCF7 cells following treatment with vehicle and 1.5 nM eribulin for 7 days. Nuclei were stained with DAPI. Images were collected from different individual experiments. (**D**) MDA-MB-231 and MCF7 cells were stained with PKH26 as detailed in *Material and Methods* and epifluorescence cellular images were collected after 7-day treatment with vehicle and 1.5 nM eribulin (left panel). PKH26 intensity was analyzed and quantified by flow cytometry (right panel). Histogram of PKH26 intensity shows that 7-day eribulin-treated cells retained label compared with vehicle-treated controls. One representative experiment is shown. (**E**) WB analysis of p21^Cip1^ protein in vehicle and 1.5 nM eribulin 7-day-treated cell lysates. Protein levels were quantified from three independent experiments. Normalization was carried out with Actin as a loading control. * *p*-value < 0.05, *** *p*-value < 0.001. (**F**) Drug-holiday experiment was performed according to the treatment scheme (upper panel) in MDA-MB-231 and MCF7 cell lines. Crystal violet staining of a representative experiment using different eribulin concentrations (left panel). Graphs show the quantification of cell viability after solubilization of crystal violet dye as detailed in *Material and Methods* (right panel).

To better understand the response of breast cancer cells to eribulin, we characterized the cells that survived treatment with an IC80 dosage of the drug (1.5 nM, empirically determined). We used MDA-MB-231 (TNBC; ER-negative) and MCF7 (luminal A type, ER+) cell lines to cover two major molecular subtypes of breast cancer. In addition, we added experiments using other TNBC cell lines: BT549, HS578T, HCC1937, plus a non-malignant cell line MCF10A. Dose–response curves for eribulin treatment were similar for MDA-MB-231 and MCF7 cell lines confirming an IC80 of 1.5 nM eribulin. In contrast, our non-malignant control MCF10A showed an IC80 of 5 nM, a higher concentration compared to cancer cell line models (Supplemental Figure S1). Although eribulin treatment of both MDA-MB-231 and MCF7 cells led to high levels of cell death, a subpopulation of large cells (10–20% of total cells) survived 7 days after initiation of the IC80 treatment (Figure 1A). The same behavior was also observed in three additional TNBC cell lines and in the non-malignant MCF10A cells (Figure 1A). Eribulin is a tubulin binder and hence, we performed tubulin and actin immunofluorescence staining to confirm its effect on the cellular cytoskeleton. As expected, the images collected showed complete disruption of the cytoskeleton, and DAPI staining confirmed the multinucleated phenotype of the cells after treatment (Figure 1B,C). Next, we studied cell proliferation in the surviving subpopulation. When cells labeled with PKH-26 dye were analyzed by either epifluorescence microscopy or flow cytometry, both cell lines showed that eribulin-treated cells retained more PKH-26 labels than vehicle-treated controls (Figure 1D), indicating slower proliferation rates. To corroborate these data, we confirmed that p21^Cip1^, a factor that promotes cell cycle arrest, is highly expressed in cells treated with eribulin compared to vehicle-treated cells (Figure 1E) [33]. Together, these results showed that the eribulin-surviving subpopulation consists of slow-cycling cells, typically referred to as drug-tolerant persister (DTP) cells. One of the most important phenotypic features of DTP cells is that they are characterized by a reversible drug-tolerance mechanism, i.e., in drug-free media DTP cells resume proliferation and reacquire drug sensitivity [34]. Given this, we treated both cell lines for 7 days with 1.5 nM eribulin to generate DTP cells, followed by either 7 days of continued eribulin treatment or 7 days of no drug (“drug holiday”; see treatment scheme in Figure 1F). Both of these treatment groups were then treated for 7 more days with either no drug or increasing eribulin concentrations. Results showed that DTP cells that were propagated for 7 more days in drug-free media (drug holiday group) were more sensitive to eribulin than DTP cells that continued to receive eribulin for 7 more days (Figure 1F). Collectively, these results show that high doses of eribulin generate a metastable DTP cell population in breast cancer cell lines. These cells are largely multinucleated, show a low proliferation rate and decreased sensitivity to eribulin, yet show a reversible behavior towards drug tolerance if the selective pressure of continuous eribulin is removed.

### 3.2. Eribulin-Induced DTP Cells Display a Specific Secretome

**Figure 2:**
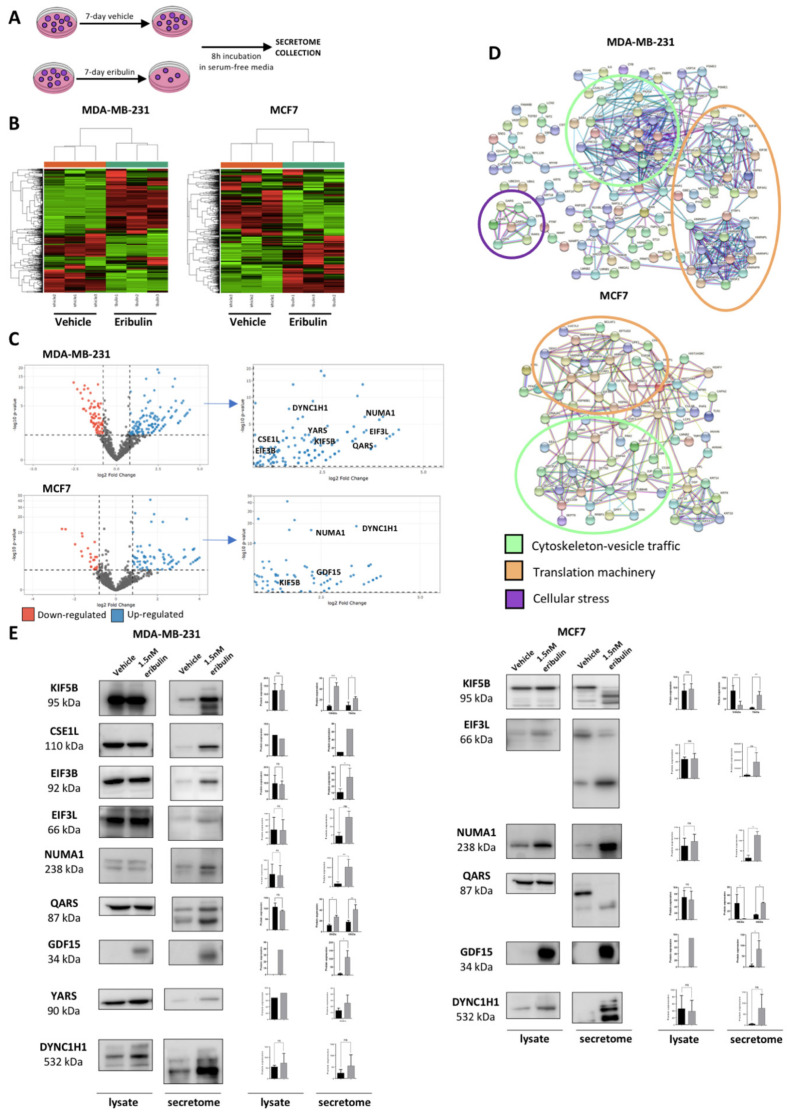
The secretome profiling of DTP cells reveals an over-secretion of a specific subfamily of biomarkers. (**A**) Schematic representation of secretome collection after a vehicle and eribulin treatment. (**B**) Heat maps representing the proteins that were significantly over-and/or down secreted, when MDA-MB-231 and MCF7 cells were treated for 7 days with vehicle or IC80 eribulin (representative heat maps of one out of three biological replicates showed in Supplemental data). Columns represent samples (per group); rows are m/z peaks (not in numerical order). (**C**) Volcano plots of the up-regulated (blue) and down-regulated (red) proteins after 7-day IC80 eribulin treatment. For each plot, the *x*-axis represents the log two-fold change (FC), and the *y*-axis represents -log 10 *p*-value. To identify statistically significant differentially secreted proteins, a Poisson statistical test was used to model our spectral count dataset. Adjusted *p*-value < 0.05, fold change > 0.8 and number spectral counts (SpC) > 4 thresholds were applied during the analysis. Enlargement of the up-regulated protein panel in the Volcano plot shows the eribulin induced secreted candidate biomarkers according to the protein subfamilies resulting from the STRING analysis (right panel). (**D**) STRING Network Analysis of over-secreted proteins after 7-day IC80 eribulin treatment showed two specific interaction nodes related to cytoskeleton-vesicle trafficking (green) and translation machinery (orange) protein subfamily. (**E**) Cell lysates (10 μg) and secretome (10 μg) were resolved by SDSPAGE and Western blotted against the different candidate biomarkers to confirm proteomic data. The figure shows a representative Western blot and the relative quantification from three biological replicates. * *p*-value < 0.05, ** *p*-value < 0.01, *** *p*-value < 0.001. GDF15 protein did not pass the established thresholds in the analysis for the MDA-MB-231 cell line, but nevertheless, Western blot analysis confirmed its secretion in this model.

DTP cells have emerged as a relevant biological entity in the therapeutic landscape of cancer patients, and a major obstacle to successfully treating cancer [12]. To identify DTP-specific biomarkers induced by eribulin, we used quantitative proteomics to profile the secretome of TNBC MDA-MB-231 and luminal A MCF7 BC cell lines after 7 days of treatment with either 1.5 nM eribulin or vehicle. Secretomes were collected after additional incubation of the cells for 8 h in serum-free media (Figure 2A). Then, after protein concentration and tryptic digestion, secretomes were analyzed by label-free quantitative proteomics (see Material and Methods). To identify statistically significant differentially secreted proteins, a Poisson statistical test was used during the statistical modeling of the proteomic data. Thresholds consisting of an adjusted *p*-value < 0.05, log_2_ fold change > 0.8 and number spectral counts (SpC) > 4 were applied during the analysis. Comparative proteomic analyses from three biological replicates (Figure 2B and Supplemental Figure S2) showed a large number of statistically significant proteins that were differentially secreted when the vehicle vs. eribulin conditions were compared (Figure 2B). We focused on the group of proteins that were over-secreted in eribulin-treated cells in order to identify biological processes that were enriched after treatment (Figure 2C). Through protein network analysis, three specific networks emerged among the eribulin-induced secreted proteins (ref STRING): one network contained proteins related to cytoskeleton-vesicle trafficking, another contained translation machinery proteins, while the third contained proteins linked to cellular stress (Figure 2D). Based on these proteomics data, a group of candidate biomarkers of eribulin response was selected; selection was made in accordance with statistical thresholds and the biological processes enriched (Figure 2D, Table 1). Next, protein expression of the candidate biomarkers identified by mass spec-based proteomics was verified by Western blot (Figure 2E). The results show that in most cases, candidate biomarkers showed biomarker potential only at the secreted level, not whole-cell or cytoplasmic levels as approximated by cell lysates. Furthermore, for some candidates including KIF5B, DYNC1H1, EIF3L and QARS, secreted proteins appear to be fragmented. Presumably, such fragmentation is due to the activation of proteolytic cascades during the onset of apoptosis induced by eribulin. In support of this explanation, and consistent with the induced stress and high level of cell death seen by treatment with high dose eribulin, both cell lines showed secretion of stress-related proteins, including aminoacyl-tRNA synthetases such as QARS and YARS, and molecular chaperones such as HSP90. One stress protein, in particular, GDF15, showed a strikingly clear on/off behavior in vehicle vs. eribulin-induced secretomes. Despite having passed the proteomic analysis statistical threshold for only one of the two cell lines (MCF7; Table 1), marked eribulin-induced secretion of GDF15 was confirmed in both cell lines by Western blotting (Figure 2E).

### 3.3. Secretomes Induced by MTAs in Breast Cancer Cells Are Linked to Their Tubulin-Binding Mechanism

**Figure 3:**
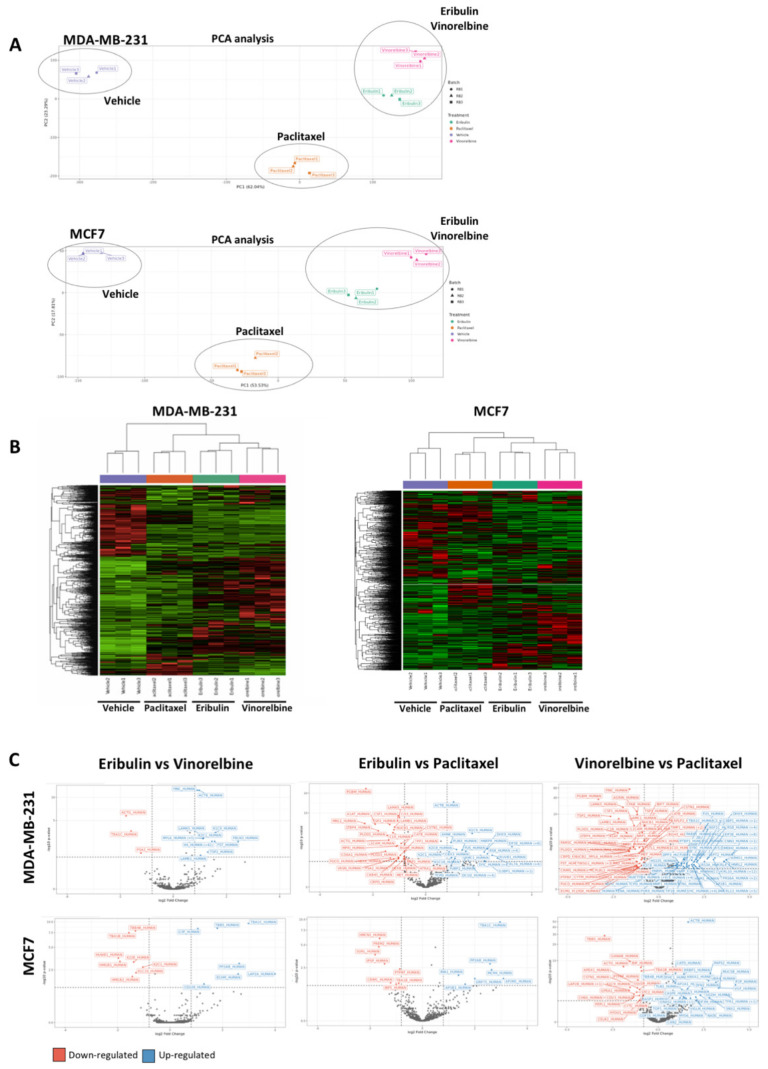
MTAs that share the same mechanism of action are characterized by a similar secretome profiling. (**A**) Representative image of the principal component analysis (PCA) of the three MTAs treatment plus vehicle (control) induced secretome. The analysis shows clear separation by treatment and a higher similarity of eribulin and vinorelbine treatment compared with paclitaxel treatment. Analysis was performed in three biological replicates (Supplemental data). (**B**) Heat maps of hierarchical clustering analysis of the different MTA treatments. The analysis confirms the clustering of eribulin and vinorelbine treatment compared with paclitaxel treatment. Analysis was performed in three biological replicates (Supplemental data). (**C**) Volcano plots of inferential analysis confirmed that differentially over-secreted proteins were obtained from the eribulin versus paclitaxel comparisons than from the eribulin versus vinorelbine one in the secretome of MDA-MB-231 and MCF7 cell-line models. Adjusted *p*-value < 0.05, fold change > 0.8 and number spectral counts (SpC) > 4 thresholds were applied during the analysis.

Microtubule targeting agents are mechanistically grouped into microtubule-stabilizing agents (MSAs) which promote the microtubule assembly, and microtubule-destabilizing agents (MDAs) which promote microtubule disassembly [18]. We hypothesized that MTAs that share similar mechanisms of action would have similar drug-induced secretomes, whereas less mechanistic similarity would result in greater secretome divergence. To test this hypothesis, we analyzed cell secretomes from MDA-MB-231 and MCF7 cell lines after 7-day treatment with eribulin, vinorelbine (both MDAs) and paclitaxel (an MSA). Unsupervised analysis of the dataset containing the three MTAs with three biological replicates each (Figure 3A,B and Supplemental Figure S3) showed that the secretome replicates obtained from cells treated with vinorelbine and eribulin were indeed grouped much closer together relative to the paclitaxel secretome profile (Figure 3A,B). Our hypothesis was further confirmed after performing inferential analysis, where more differentially over-secreted proteins were identified when comparing MDAs (eribulin or vinorelbine) versus MSAs (paclitaxel) than when comparing MDAs among themselves (eribulin versus vinorelbine) (Figure 3C). These data show that the two MTAs that bind to approximately the same site on β-tubulin (eribulin and vinorelbine), thus sharing a similar mechanism of action, also have more similar secretomes, whereas the secretome of the MSA paclitaxel shows a much greater divergence from the other two.

### 3.4. GDF15 Secretion Is Strongly Associated with the DTP State

**Figure 4:**
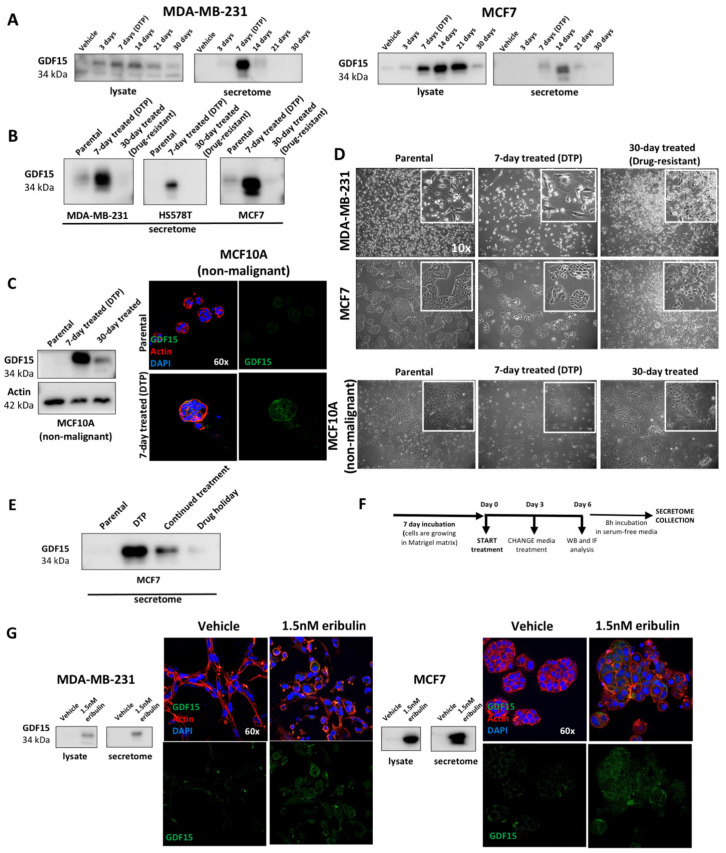

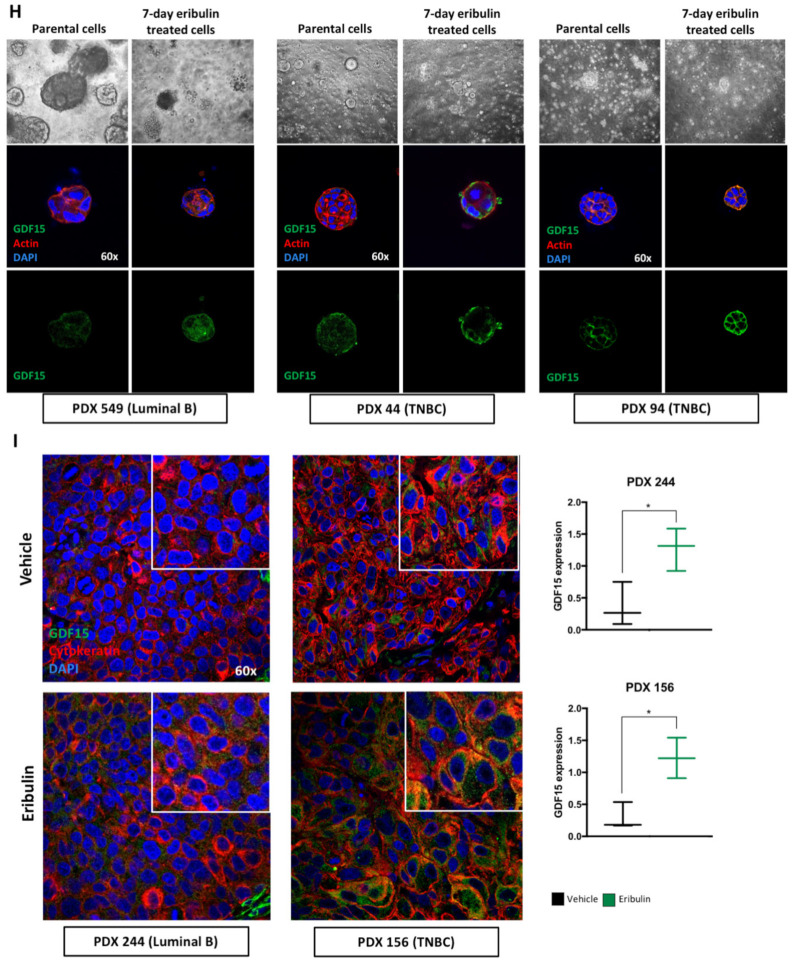
GDF15 secretion is strongly associated with DTP cells. (**A**) WB analysis of GDF15 protein in the vehicle and 1.5 nM eribulin cell lysates and secretome collected at different time points during a 30-day treatment in MDA-MB-231 and MCF7 cell lines. (**B**) WB analysis of GDF15 protein in secretome collected from 7-day vehicle (parental), 7-day 1.5 nM eribulin (DTP) and 30-day 1.5 nM eribulin (drug-resistant)-treated MDA-MB-231 and MCF7 cells. (**C**) Left: WB analysis of GDF15 protein in secretome collected from 7-day vehicle (parental), 7-day 1.5 nM eribulin (DTP) and 30-day 1.5 nM eribulin (Non-fully drug-resistant)-treated MCF10A (non-malignant) cells. Right: Representative confocal images of GDF15 (green) and Actin (red) immunofluorescence in 3D cell-cultured MCF10A (non-malignant) cells following treatment with vehicle and 1.5 nM eribulin for 7 days. Nuclei were stained with DAPI. One representative experiment is shown. (**D**) Representative cell pictures of the different cell states: parental, DTP and drug-resistant/non-fully drug-resistant. (**E**) WB analysis of GDF15 in the secretome collected from MCF7 cells in the following treatment conditions: 7-day vehicle (parental) treated, 7-day 1.5 nM eribulin (DTP) treated, 7-day 1.5 nM eribulin-treated cells pre-treated for 7 days more with eribulin (Continued treatment) and 7-day 1.5 nM eribulin-treated cells propagated for 7 days in drug-free media (Drug holiday). (**F**) Schematic representation of eribulin treatment and secretome collection in 3D cell culture. (**G**) WB analysis of GDF15 protein in secretome collected from 3D cell-cultured MDA-MB-231 and MCF7 following treatment with vehicle and 1.5 nM eribulin for 7 days. Representative confocal images of GDF15 (green) and Actin (red) immunofluorescence in 3D cell-cultured MDA-MB-231 and MCF7 cells following treatment with vehicle and 1.5 nM eribulin for 7 days. Nuclei were stained with DAPI. One representative experiment is shown. (**H**) Cellular images (upper panel) and confocal images of GDF15 (green) and Actin (Red) (lower panel) in two representative breast cancer PDX models treated for 7 days with vehicle and 1.5 nM eribulin in a 3D cell culture system. (**I**) Representative confocal images of GDF15 (green) staining in breast cancer tissues derived by PDX tumor cells treated with eribulin in vivo for a month. Staining for human-cytokeratin (red) allowed for discrimination of human epithelial cells from other cell components. Nuclei were stained with DAPI. Graphs show quantification of GDF15 (green) staining intensity normalized to number of cells (DAPI discrimination. Minimum of 150 cells in three different regions of interest). * *p*-value < 0.05.

Over-secretion of GDF15 induced by eribulin in our studies of breast cancer cell lines led us to investigate its potential as a pharmacodynamic (PD) response biomarker for eribulin, in addition to its potential utility as a specific biomarker for DTP cell generation. To test this hypothesis, we performed kinetic studies on the expression and secretion of GDF15. First, MDA-MB-231 and MCF7 cell lines were treated for one month with 1.5 nM of eribulin. As before, cells were initially sensitive to eribulin, with approximately 85% of cells dying in the first 4 days of treatment, followed by about three weeks during which surviving cells remained in a slow-cycling DTP state. At this point, cells started to proliferate again in the presence of the drug, suggesting that a stable mechanism of drug resistance had been established. In parallel with monitoring drug sensitivity, we also measured GDF15 levels in both lysates and secretomes each week during the month-long treatment. Intriguingly, GDF15 expression/secretion was detectable only in the DTP state (Figure 4A). Moreover, neither sensitive cells (parental) nor fully resistant cells (end-stage cells that can proliferate in the presence of 1.5 nM eribulin) expressed or secreted GDF15. This result was also confirmed in another TNBC cell line, Hs578T cells (Figure 4B), and in the non-malignant MCF10A cells (Figure 4C). Cellular images collected at different cell states visually show that DTP cells are morphologically different than both parental and end-stage drug-resistant cells (Figure 4D). MDA-MB-231 and MCF7 cells regain proliferation after 3 weeks of eribulin treatment and hence become fully resistant to eribulin. In contrast, the non-malignant model shows no proliferation after 3 weeks of treatment. Concomitant to the cell quiescence, MCF10A cells still express GDF15 after 30 days of treatment (Figure 4C,D). These results confirm that GDF15 is a marker in DTP cells (7-day treatment), and only when cells resume proliferation after becoming drug-resistant do they lose GDF15 expression. Interestingly, DTP cells propagated for 7 days in drug-free media, where they regain sensitivity to eribulin treatment (see Figure 1F), and showed lower levels of GDF15 secretion compared to cells that continued to receive eribulin (Figure 4E). Together, these results establish a specific correlation between GDF15 secretion and the DTP state.

As further confirmation, we validated GDF15 expression and secretion in a 3D cell culture setting. MDA-MB-231 and MCF7 cells were grown in Matrigel and treated with eribulin for 7 days as described above for the 2D culture setting (experimental scheme Figure 4F). We then confirmed that 1.5 nM eribulin still had the same cytotoxic and cytoskeleton-disrupting effects as previously seen in the 2D-cell-culture setting (Supplemental Figure S4A,B). Finally, we showed that MDA-MB-231 and MCF7 cells grown in 3D expressed and secreted GDF15 after 7-day eribulin treatment (Figure 4G). 

Additional validation of GDF15 expression and secretion as a potential biomarker was performed in breast cancer patient-derived xenograft organoids (PDXO). For these studies, we selected three PDX models belonging to the same breast cancer subtypes represented by the cell lines used in the 2D and 3D studies above, specifically TNBC and luminal subtypes. We grew PDX cells in a 3D Matrigel matrix and performed a 7-day eribulin treatment according to the same procedures described above. GDF15 immunostaining of PDXO after eribulin treatment (Figure 4H, upper and lower panels) supported the previous results from cell lines both establishing GDF15 as a biomarker of response to eribulin as well as a specific marker for DTP cell generation. 

Finally, we performed the GDF15 immunostaining on tissues from breast cancer PDXs treated in vivo with eribulin. Briefly, mice were treated intravenously with eribulin (0.1 mg/kg in PBS) for 4 weekly Q2Dx3 cycles with PDX tumors being collected after the fourth cycle. In agreement with predictions based on 2D and 3D cell lines and PDXO results, PDX tumors from mice treated with eribulin expressed more GDF15 than samples from vehicle-treated mice (Figure 4I). 

### 3.5. GDF15 Is Required for Maintenance of the DTP State in Cells Responding to Eribulin

**Figure 5:**
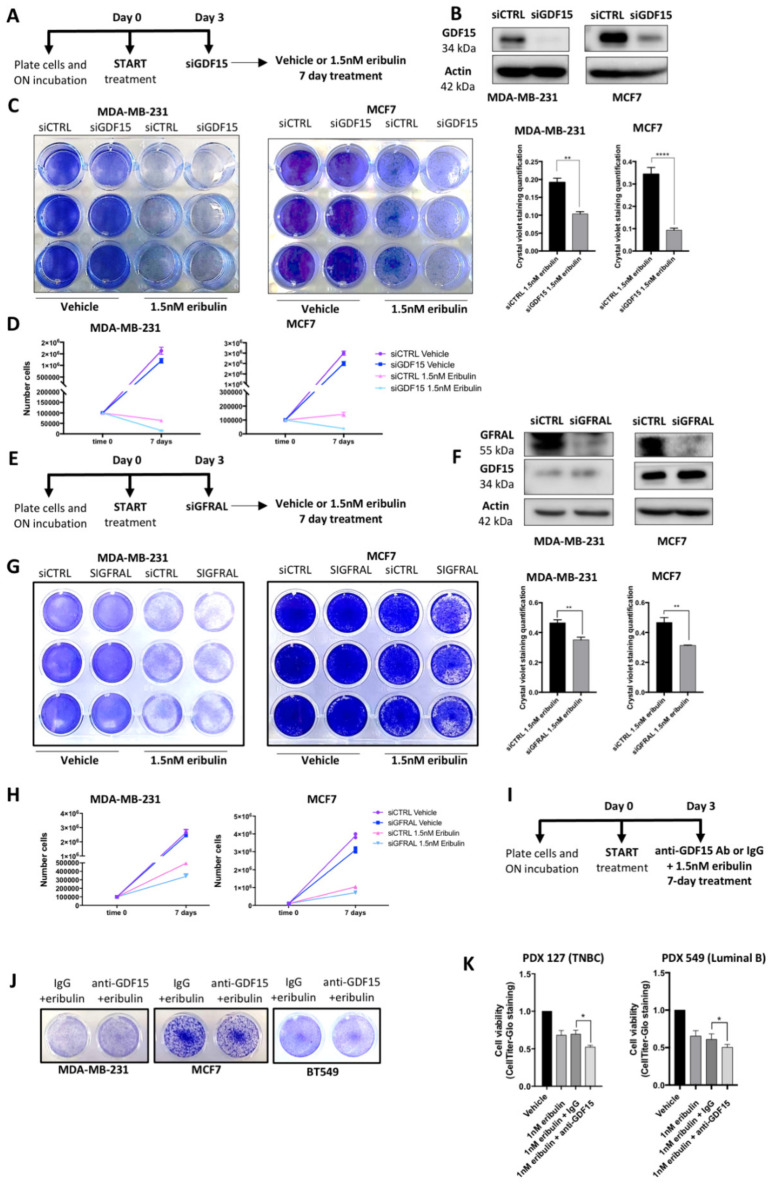
GDF15 contributes to the maintenance of the DTP state in cells responding to eribulin. (**A**) Schematic representation of siGDF15 experiment and following 7-day eribulin treatment. (**B**) WB analysis of GDF15 protein in siCTRL and siGDF15 cells. Normalization was carried out with Actin as a loading control. (**C**) Representative image of crystal violet staining in siCTRL and siGDF15 cells treated for 7 days with the vehicle and 1.5 nM eribulin treatment. Graphs (right panel) show the quantification of cell viability in siGDF15 cells during eribulin treatment compared with siCTRL cells. ** *p*-value < 0.01, **** *p*-value < 0.0001. (**D**) Cell-counting experiment performed in siCTRL and siGDF15 cells after a 7-day vehicle and 1.5 nM eribulin treatment. The graphs show higher sensitivity to eribulin of siGDF15 cells compared with siCTRL cells. The graph for each cell line shows the mean viable cell number ± SD calculated from three independent experiments. (**E**) Schematic representation of siGFRAL experiment and following 7-day eribulin treatment. (**F**) WB analysis of GFRAL and GDF15 protein in siCTRL and siGFRAL cells. Normalization was carried out with Actin as a loading control. (**G**) Representative image of crystal violet staining in siCTRL and siGFRAL cells treated for 7 days with the vehicle and 1.5 nM eribulin treatment. Graphs (right panel) show the quantification of cell viability in siGFRAL cells during eribulin treatment compared with siCTRL cells. ** *p*-value < 0.01. (**H**) Cell-counting experiment performed in siCTRL and siGFRAL cells after a 7-day vehicle and 1.5 nM eribulin treatment. The graphs show higher sensitivity to eribulin of siGFRAL cells compared with siCTRL cells. The graph for each cell line shows the mean viable cell number ± SD calculated from three independent experiments. (**I**) Schematic representation of anti-GDF15 Ab treatment in combination with 1.5 nM eribulin for 7 days. (**J**) Representative images of crystal violet staining in MDA-MB-231, MCF7 and BT549 cell lines treated for 7 days with vehicle, eribulin, eribulin + IgG and eribulin + anti-GDF15 Ab. * *p*-value < 0.05. (**K**) Graphs of cell viability quantification using CellTiter-Glo staining in PDXO treated for 7 days with vehicle, eribulin, eribulin + IgG and eribulin + anti-GDF15 Ab.

Finally, we explored the possibility that GDF15 not only correlates with the DTP state but also plays a functional role in establishing and maintaining tolerance to eribulin in DTP cells. To do this, we first down-regulated GDF15 expression by siRNA in both MCF7 and MDA-MB-231 cells (Figure 5A,B). Importantly, siGDF15 cells showed a higher sensitivity to eribulin compared to siCTRL cells, even though the proliferation of both siCTRL-treated and siGDF15-treated cells in the absence of eribulin was unaffected (Figure 5C,D). These results establish that GDF15 is not only a marker for the generation of the DTP state following eribulin treatment, but actually plays a functional role in maintaining it. Since our results showed that eribulin induced both expression and secretion of GDF15 in DTP cells, we hypothesized that the connection between GDF15 and eribulin sensitivity depended directly on secreted GDF15. To test this, we down-regulated GFRAL (receptor of GDF15) expression by siRNA in both MCF7 and MDA-MB-231 cells (Figure 5E,F). Importantly, GFRAL knockdown by siGFRAL led to enhanced responses to eribulin relative to cells receiving siCTRL, supporting the hypothesis that secreted GDF15 plays a functional role in establishing and maintaining the eribulin-tolerant DTP state (Figure 5G,H).

Finally, with these data in hand, we reasoned that an effective GDF15 inhibitor could potentially be used in a clinical setting to target DTP cells and thus maintain the initial sensitivity to eribulin. To test this possibility, we performed a combination treatment of eribulin plus an anti-GDF15 antibody in both MDA-MB-231 and MCF7 cell lines (Figure 5I). Crystal violet staining showed that blocking secreted GDF15 with the antibody effectively reduced the residual DTP cell pool in presence of eribulin treatment (Figure 5J), further confirming a direct role for GDF15 in establishing and maintaining the DTP state. Furthermore, the difference in the response of the two cell lines to the eribulin and anti-GDF15 antibody combination suggests that the degree of benefit of inhibiting GDF15 depends on the level of GDF15 secretion. Since there is always a greater up-regulation of GDF15 in MCF7 compared to MDA-MB-231 cells, we looked at another TNBC cell line, BT549, which shows higher GDF15 secretion compared to MDA-MB-231 upon eribulin treatment (Supplemental Figure S5A,B). As hypothesized, results obtained with BT549 cells are closer to those obtained with MCF7. Together, these data suggest that cells with the higher secretion of GDF15 are more dependent on it for the establishment and maintenance of the DTP state, and thus show a higher synergy between eribulin and the anti-GDF15 antibody (Supplemental Figure S5C). Finally, to extend these conclusions beyond the limitations of cell lines, we validated the effects of combination treatment of anti-GDF15 antibody plus eribulin in both TNBC and luminal 3D PDXO models. Quantification of cell viability by CellTiter in these 3D cultures showed that cells treated with the drug combination are more sensitive to eribulin treatment (Figure 5K). Collectively, these results suggest the possibility that combination treatment with eribulin plus GDF15 inhibitors could both enhance response and forestall the development of resistance in the breast cancer setting.

## 4. Discussion

Until recently, tumor sensitivity to cancer drugs and acquired drug resistance were usually seen as a binary switch. In this simplistic view, tumor cells would first die due to the action of the drug and then, eventually, the residual tumor would gain or become enriched with a mutation that would cause the resumption of tumor growth [32,35,36]. Recently, however, the concept of drug *tolerance*, which lies between drug sensitivity and drug resistance, has changed the paradigm of how tumor cells respond to cancer drugs. Drug tolerance is induced by therapeutic stress through non-genetic reprogramming that then leads to a reversible quiescent state [37]. In this work, we focused on the proteomic characterization of breast cancer drug-tolerant cells that survive treatment with eribulin. First, we proved that when treated with a high dose of eribulin, breast cancer cells enter into a reversible slow-cycling state, known as the drug-tolerant persister (DTP) state. Then, we performed secretome proteomic profiling of breast cancer-DTPs to identify candidate pharmacodynamic biomarkers that could inform the action of eribulin in breast cancer patients. The DTP secretome induced by eribulin showed over-secretion of different groups of proteins related to the cytoskeleton, vesicle trafficking, protein translation and cellular stress, in agreement with the biological effects of a tubulin targeting agent. Interestingly, however, two cell lines belonging to different BC molecular subtypes showed similar eribulin-induced secretomes. Moreover, our results suggest that the secretome profiles of DTPs induced by different MTAs in breast cancer cells are linked to their specific tubulin-binding modes and mechanisms [17,38]. 

One protein over-secreted in DTP cells, GDF15, attracted our particular attention since its expression and secretion in parental cells were undetectable. The existing literature indicates that GDF15 is related to a wide range of very different biological processes in human biology. For instance, GDF15 is strongly up-regulated during pregnancy and its levels remain high in neonates until they are about 2 years old [39]. In addition, elevated serum levels of GDF15 correlate with diabetes, chronic inflammation, infections, as well as both cardiovascular and renal diseases [23]. The mechanistic link between the roles of GDF15 in these various conditions is not well known. However, current knowledge suggests that induction of GDF15 expression is linked to either physiological or pathological stress related to energy balance [29]. The recent discovery of the receptor for GDF15, the previous orphan receptor GFRAL [26], will undoubtedly contribute to our understanding of the mechanisms of action of GDF15.

In cancer, GDF15 is elevated in the advanced stages of several cancer types, where it participates in the induction of cachexia affecting patients with late-stage cancer [40,41,42]. Different studies have shown higher expression of GDF15 mRNA and protein in cancer biopsies compared to their corresponding normal tissues [43,44]. Our results, showing that GDF15 is associated with the DTP state induced by eribulin, do not immediately connect with any known biology mediated by GDF15. However, a recent report showed that levels of circulating GDF15 correlated with chemoresistance of ovarian cancer patients to platinum therapy [45]. In light of this, our in vitro results with cell lines, PDXO and in vivo results with PDX-derived tumor tissues offer a more nuanced picture: GDF15 is a pharmacodynamic response biomarker of eribulin, even while it is also associated with the transitory DTP state between drug sensitivity and full resistance. We thus believe that the concept of drug tolerance may help solve the question of whether GDF15 is a biomarker of response or resistance; our results suggest it is both. 

The down-regulation of GDF15 in cells that achieved full drug resistance, and resume proliferation, confirms that GDF15 is a stress response protein. As stated above, different cellular stresses induce the expression of GDF15 to mitigate different acute insults including infection, injuries and cancer chemotherapy. In these cases, the role of GDF15 is to block cell proliferation, protect the damaged cells and tissues, and restore homeostasis [23,26]. In our experimental framework, a group of tumor cells (DTPs) are able to survive a high dose of eribulin by up-regulating the expression of GDF15. Eventually, these DTP adapt to eribulin and they are able to proliferate in the presence of the drug. We cannot rule out that the down-regulation of GDF15 only correlates with acquired resistance to eribulin instead of being the cause of it. However, our results, showing that targeting GDF15 blocks the survival of DTP cells, favor the hypothesis that GDF15 is mechanistically linked to the proliferative Stop/Go phenotype shown by DTP and fully resistant cells, respectively. When we translate this hypothesis into the clinical setting, we predict that the down-regulation of GDF15 expression in a tumor responding to eribulin would anticipate the progression of the disease.

One of the most striking results of our studies is that GDF15 not only correlates with the DTP state induced by eribulin but is also required for the survival of DTP cells: silencing of both GDF15 and its receptor GFRAL led to significant increases in eribulin sensitivity in both cell lines tested. Importantly, control experiments established that modulation of GDF15 and GFRAL did not affect cell proliferation per se; therefore, up-regulation of GDF15 is required for the viability of DTP cells. Moreover, we confirmed that actual secretion of GDF15, and not just protein expression, mediates the survival phenotype in DTP cells, since GFRAL silencing had the same effect as GDF15 silencing. Intriguingly, it has recently been reported that a GDF15-neutralizing antibody reverses anorexia and body weight loss and promotes survival in mouse tumor models [46]. Additionally, a recent study showed that treatment with a GDF15-neutralizing antibody in combination with cisplatin improves survival in a mouse tumor model [47]. Our GDF15/GFRAL silencing experiments together with the aforementioned GDF15-neutralizing antibody studies led us to test combination therapy with eribulin plus an anti-GDF15 antibody in our experimental models. Results confirmed our suspicions that interfering with GDF15 would enhance response to eribulin: significantly increased cell death was seen when breast cancer cells were treated with the combination of anti-GDF15 antibody plus eribulin as compared to the combination using a nonspecific control IgG. Similar results to the cell line studies were seen when treating breast cancer-derived PDXO with the same combinations, supporting the idea that GDF15 neutralization in combination with drug treatment may hold considerable potential as an effective therapeutic approach for breast cancer. Perhaps most excitingly, our work suggests that combining an anti-GDF15 antibody with chemotherapy could improve the overall survival of patients by the dual mechanisms of decreasing cachexia and either killing or preventing the development of DTPs during chemotherapy. Thus, our data in the breast cancer space suggest that GDF15 should be considered as a possible therapeutic co-target to eradicate or prevent the development of DTP cells during treatment with eribulin in both TNBC and luminal breast cancer subtypes. Intriguingly, our discovery of links between GDF15 and DTP cells as a result of eribulin treatment may extend to other drugs as well: preliminary data with several other drugs including metformin, doxorubicin, carboplatin and paclitaxel point to up-regulation of GDF15 by these drugs as well, perhaps also associated with DTP states (Supplemental Figure S6). While further work will be required to confirm this, these preliminary results suggest that the paradigms established here with eribulin may also hold generally true for at least some other chemotherapy drugs.

## 5. Conclusions

We have identified the secretion of GDF15 as a pharmacodynamic response biomarker of eribulin treatment by focusing on the proteomic characterization of BC drug-tolerant cells that survive treatment with eribulin. Intriguingly, GDF15 is also functionally associated with the transitory DTP state between drug sensitivity and full resistance. GDF15/GFRAL silencing experiments showed that GDF15 not only correlates with the DTP state induced by eribulin but it is also required for the survival of DTP cells. These results led us to test a combination therapy with eribulin plus an anti-GDF15 antibody in our experimental models confirming our hypothesis that interfering with GDF15 would enhance response to eribulin. Thus, our results support the conclusion that GDF15 should be considered as a possible therapeutic co-target to eradicate or prevent the development of DTP cells during treatment with eribulin in both TNBC and luminal breast cancer subtypes. More generally, the combination of cytotoxic agents with targeted agents aimed specifically at DTP cells may be an effective therapeutic approach to improve the treatment outcome for cancer patients.

## Figures and Tables

**Table 1:** List of eribulin-induced secreted candidate biomarkers. Representative analysis of one out of three biological replicates of spectral count data after exporting it from Scaffold software. Adjusted *p*-value < 0.05, fold change > 0.8 and number of spectral counts (SpC) > 4 thresholds were applied during the analysis. (*) Spectral counts of GDF15 protein in the MDA-MB-231 cell line as evidence that it did not appear in the volcano plot for the thresholds considered in the analysis as described in *Material and Methods*.

**MDA-MB-231 Up-Regulated Proteins**				
**Prot, Nm**	**Eribulin**	**Vehicle**	**Log2FC**	**adj *p*-Value**
DYNC1H1	32.1	4.7	2.71	7.54 × 10^−42^
KIF5B	9.7	1.8	2.37	1.90 × 10^−11^
CSE1L	22.1	8.l	1.374	2.52 × 10^−12^
EIF3B	15.3	5.1	1.513	4.49 × 10^−10^
EIF3L	6.8	0.3	4.273	8.34 × 10^−14^
NUMA1	7.4	0.4	3.994	2.13 × 10^−14^
QARS	5.9	0.3	4.071	9.42 × 10^−12^
YARS	5.3	0.7	2.927	2.81 × 10^−8^
GDF15 (*)	2.9	0.2	3.32	4.85 × 10^−2^
**MCF7 Up-Regulated Proteins**				
**Prot, Nm**	**Eribulin**	**Vehicle**	**Log2FC**	**adj *p*-Value**
DYNC1H1	41.8	4.2	3.149	4.59 × 10^−61^
KIFSB	14.8	4.2	1.65	2.99 × 10^−10^
EIF3L	6.9	3.6	0.7968	0.0285
NUMA1	49.7	5.9	2.917	5.16 × 10^−67^
QARS	4.8	0.6	2.946	5.14 × 10^−67^
GDF15	10.6	1.6	2.602	5.94 × 10^−13^

## Data Availability

The data presented in this study are available on request from the corresponding author. Breast cancer cell lines MDA-MB-231, MCF7, HS578T, BT549, HCC1937 and MCF10A were purchased from the American Type Culture Collection (ATCC).

## Supplementary Materials

The following supporting information can be downloaded at: https://www.mdpi.com/article/10.3390/cancers14102562/s1, Figure S1. Eribulin dose response curve in MDA-MB-231, MCF7 and MCF10A cells. Representative images of MDA-MB-231, MCF7 and MCF10A cells following treatment with indicated dose of eribulin for 7 days. Figure S2. Heat maps and Volcano plots representing the proteins that were significantly over-and/or down secreted in the second and third biological replicates. Figure S3. PCA analysis and heat maps of hierarchical clustering analysis of the different MTA treatments in the second and third biological replicates. Figure S4. Representative images of 3D cell-cultured MDA-MB-231 and MCF7 cells following treatment with indicated dose of eribulin for 7 days. Figure S5. WB analysis of GDF15 protein in siCTRL and siGDF15 cells in different breast cancer cell lines. Representative image of crystal violet staining in siCTRL and siGDF15 BT549 cells treated for 7 days with vehicle and 1.5 nM eribulin treatment. Graphs of cell viability quantification using CellTiter staining in MDA-MB-231, MCF7 and BT549 cell lines treated for 7 days with vehicle, eribulin, eribulin + IgG and eribulin + anti-GDF15 Ab. Figure S6. WB analysis of GDF15 protein in cell lysates and secretomes collected after 72 h of treatment with different drugs: eribulin, metformin, doxorubicin, carboplatin and paclitaxel. *Uncropped blots*.
