# Supplementary material for: GDF15 Is an Eribulin Response Biomarker also Required for Survival of DTP Breast Cancer Cells
Source: Cancers (Basel). 2022 May 23;14(10):2562. doi: 10.3390/cancers14102562 (PMC9139899; doi:10.3390/cancers14102562)
Supplement: Supplementary file 1 [file cancers-14-02562-s001.zip › cancers-1707203/cancers-1707203 - sup.pdf]

Supplemental Figures

Figure S1. Eribulin dose response curve in MDA-MB-231 and MCF7 cell lines (crystal violet staining quantification) and MCF10A cells (cell-counting). Representative images of MDA-MB-231, MCF7 and MCF10A cells following treatment with indicated dose of eribulin for 7 days. Images taken at 10x magnification.

Figure S2. Heat maps **(A)** and Volcano plots **(B)** representing the proteins that were significantly over-and/or down secreted in the second and third biological replicates.

Figure S3. PCA analysis **(A)** and heat maps of hierarchical clustering analysis **(B)** of the different MTA treatments in the second and third biological replicates.

Figure S4. (A) Representative images of 3D cell-cultured MDA-MB-231 and MCF7 cells following treatment with indicated dose of eribulin for 7 days. Images taken at 10x magnification. **(B)** Representative confocal images of Actin (red) immunofluorescence in 3D cell-cultured MDA-MB-231 and MCF7 cells following treatment with vehicle and 1.5nM eribulin for 7 days. Nuclei were stained with DAPI.

Figure S5. (A) WB analysis of GDF15 protein in siCTRL and siGDF15 cells in different breast cancer cell lines. Normalization was done with Actin as a loading control. **(B)** Representative image of crystal violet staining in siCTRL and siGDF15 BT549 cells treated for 7 days with vehicle and 1.5nM eribulin treatment. **(C)** Graphs of cell viability quantification using CellTiter staining in MDA-MB-231, MCF7 and BT549 cell lines treated for 7 days with vehicle, eribulin, eribulin + IgG and eribulin + anti-GDF15 Ab.

Figure S6. WB analysis of GDF15 protein in cell lysates and secretomes collected after 72 hours of treatment with different drugs: eribulin, metformin, doxorubicin, carboplatin and paclitaxel.

Figure S1

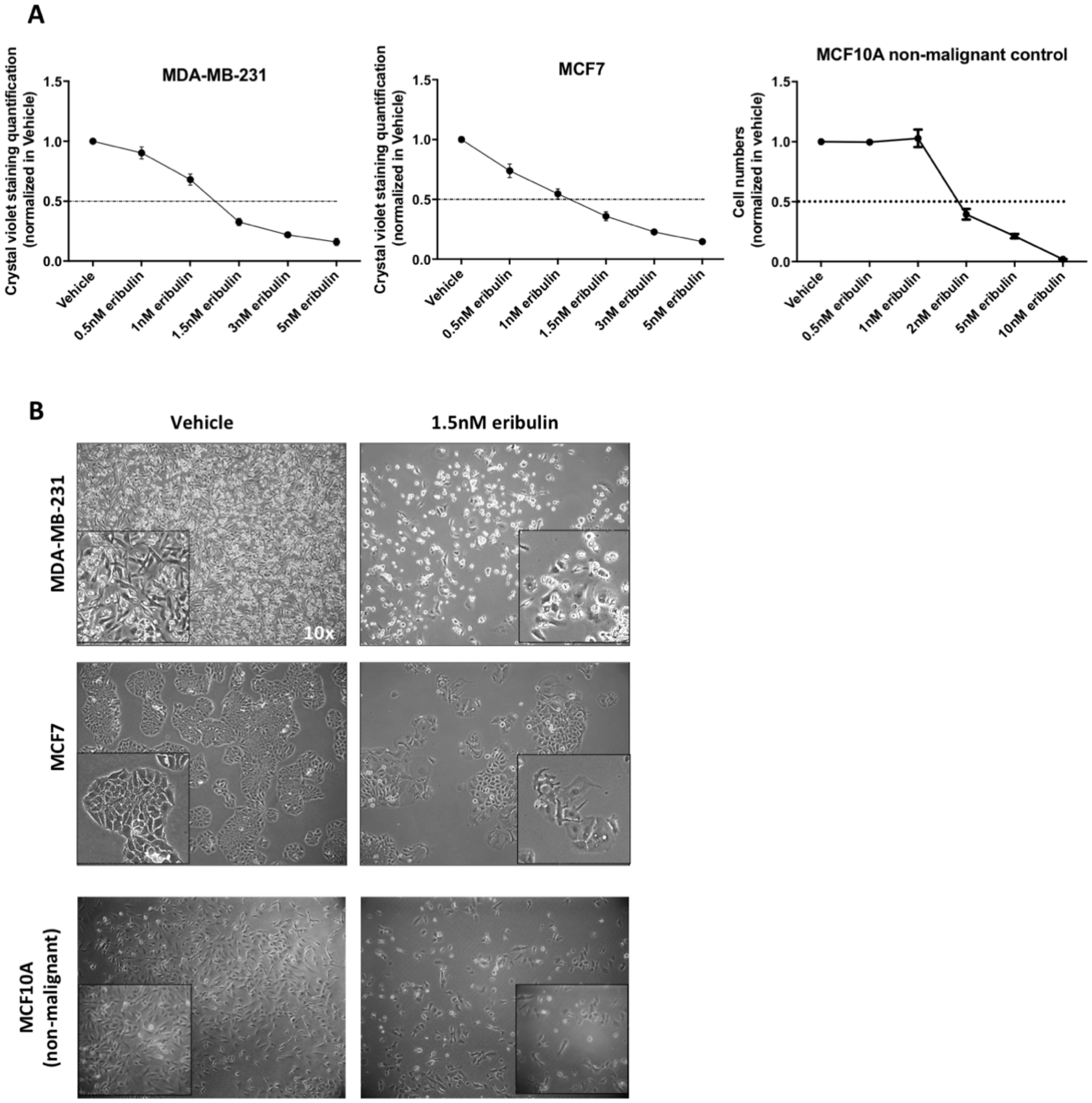
A
MDA-MB-231
Crystal violet staining quantification (normalized in Vehicle)
1.5
1.0
0.5
0.0
Vehicle
0.5nM eribulin
1nM eribulin
1.5nM eribulin
3nM eribulin
5nM eribulin
MCF7
Crystal violet staining quantification (normalized in Vehicle)
1.5
1.0
0.5
0.0
Vehicle
0.5nM eribulin
1nM eribulin
1.5nM eribulin
3nM eribulin
5nM eribulin
MCF10A non-malignant control
Cell numbers (normalized in vehicle)
1.5
1.0
0.5
0.0
Vehicle
0.5nM eribulin
1nM eribulin
2nM eribulin
5nM eribulin
10nM eribulin
B
Vehicle
1.5nM eribulin
MDA-MB-231
10x
MCF7
MCF10A (non-malignant)

Figure S2

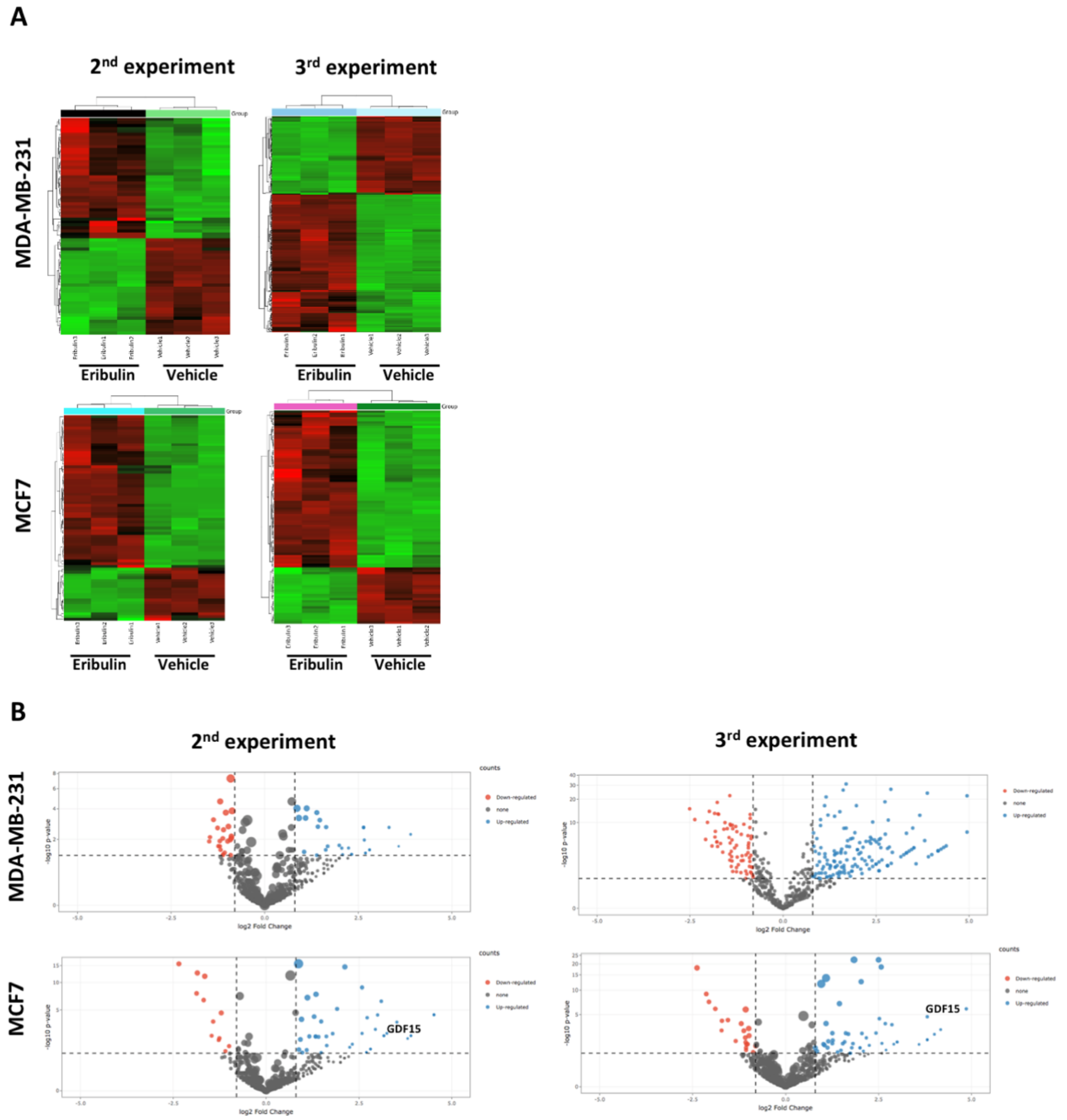
A
2nd experiment
3rd experiment
MDA-MB-231
MCF7
Eribulin
Vehicle
Eribulin
Vehicle
Eribulin
Vehicle
Eribulin
Vehicle
B
2nd experiment
3rd experiment
MDA-MB-231
MCF7
GDF15
GDF15
log2 Fold Change
-log10 p-value
counts
Down-regulated
none
Up-regulated

Figure S3

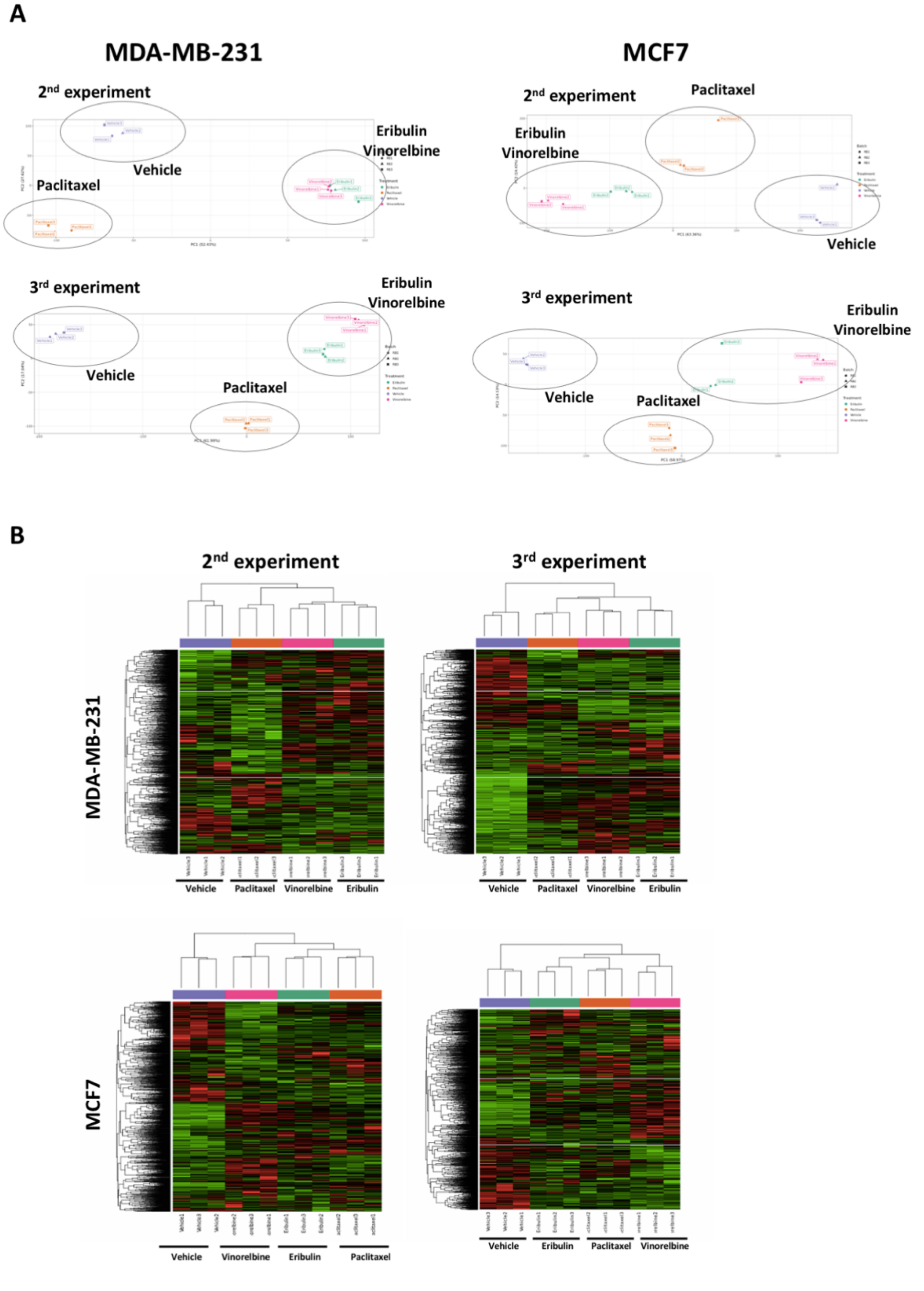
A
MDA-MB-231
MCF7
2nd experiment
Vehicle
Paclitaxel
Eribulin
Vinorelbine
3rd experiment
B
MDA-MB-231
MCF7
Vehicle
Paclitaxel
Vinorelbine
Eribulin

Figure S4

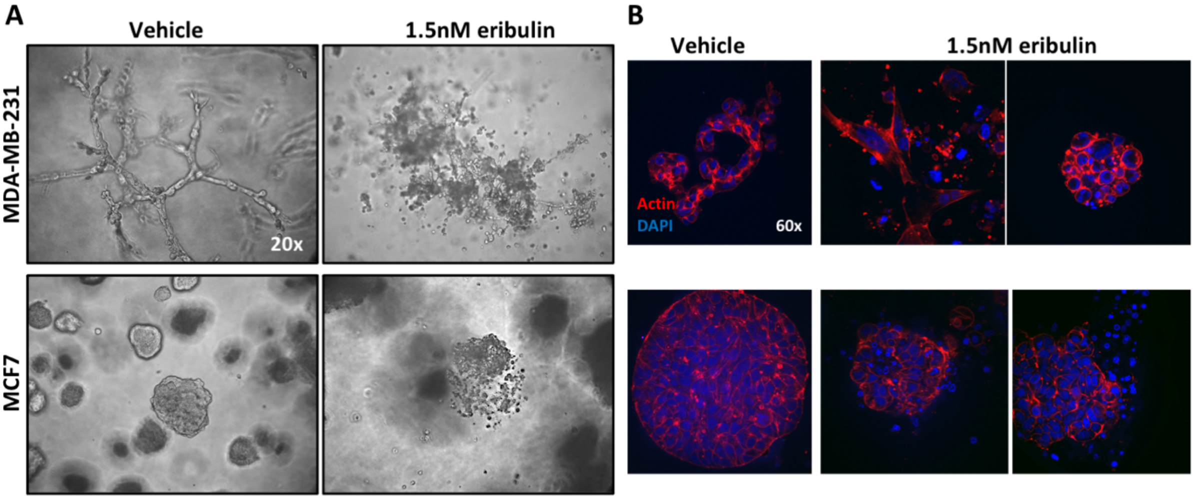
A
Vehicle
1.5nM eribulin
MDA-MB-231
MCF7
20x
B
Vehicle
1.5nM eribulin
Actin
DAPI
60x

Figure S5

A

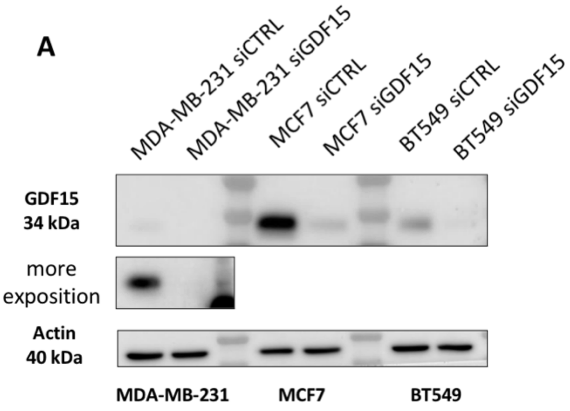

B

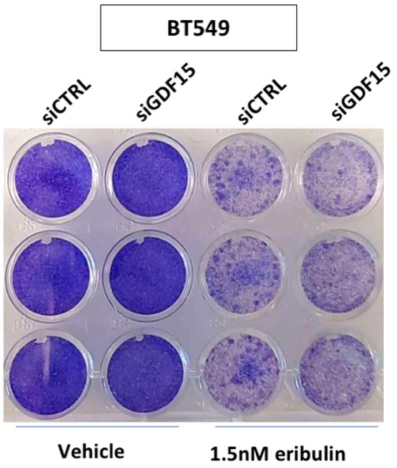

C

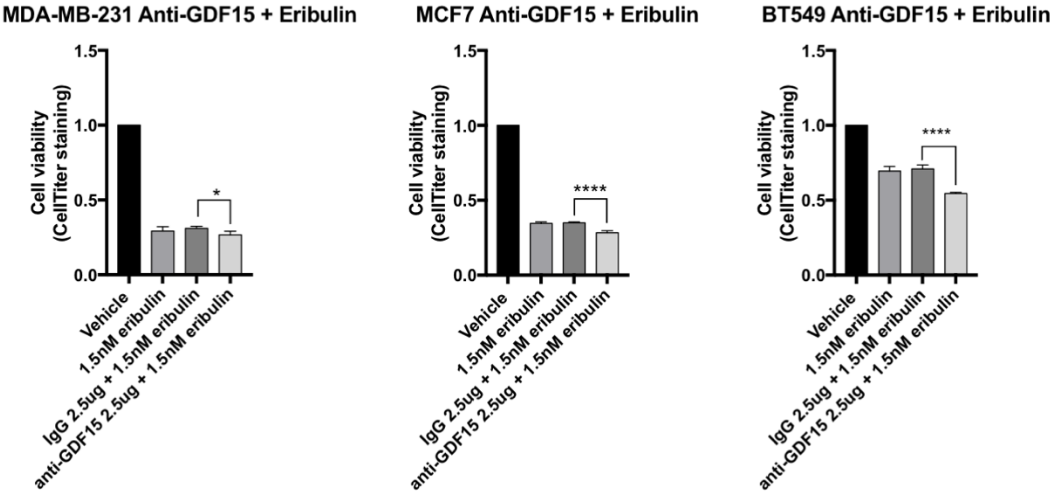

Figure S6

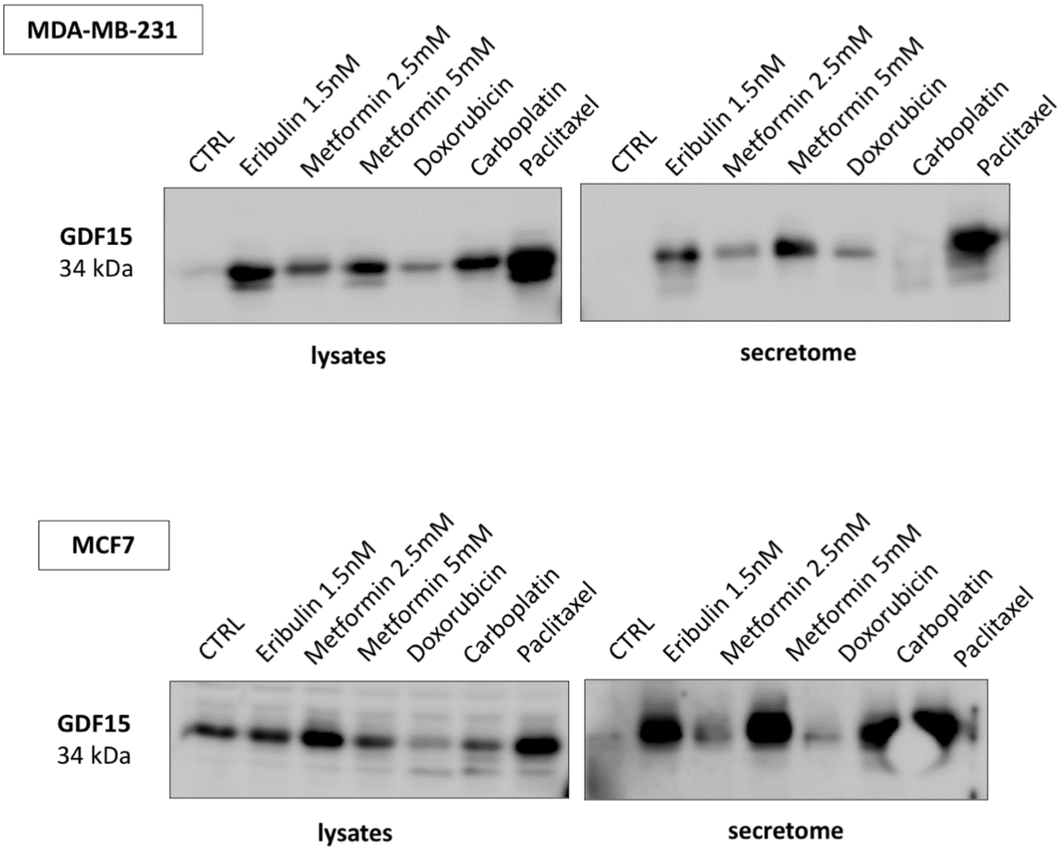
MDA-MB-231
CTRL
Eribulin 1.5nM
Metformin 2.5mM
Metformin 5mM
Doxorubicin
Carboplatin
Paclitaxel
GDF15
34 kDa
lysates
secretome
MCF7
GDF15
34 kDa
lysates
secretome

Uncropped blots

Figure 1 E

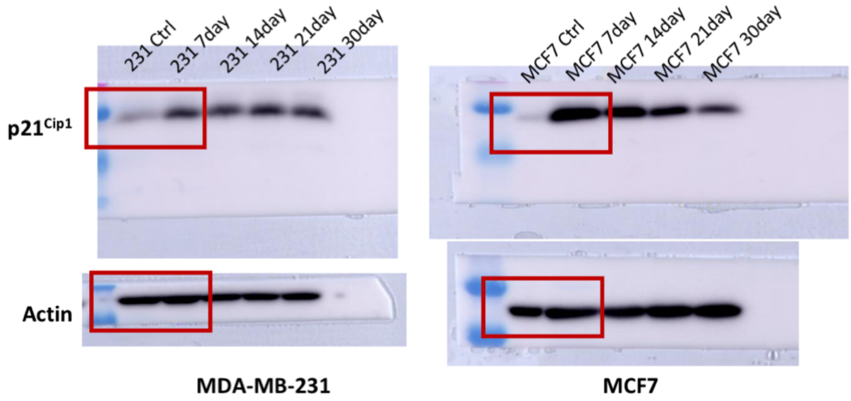

Figure 2 E – MDA-MB-231

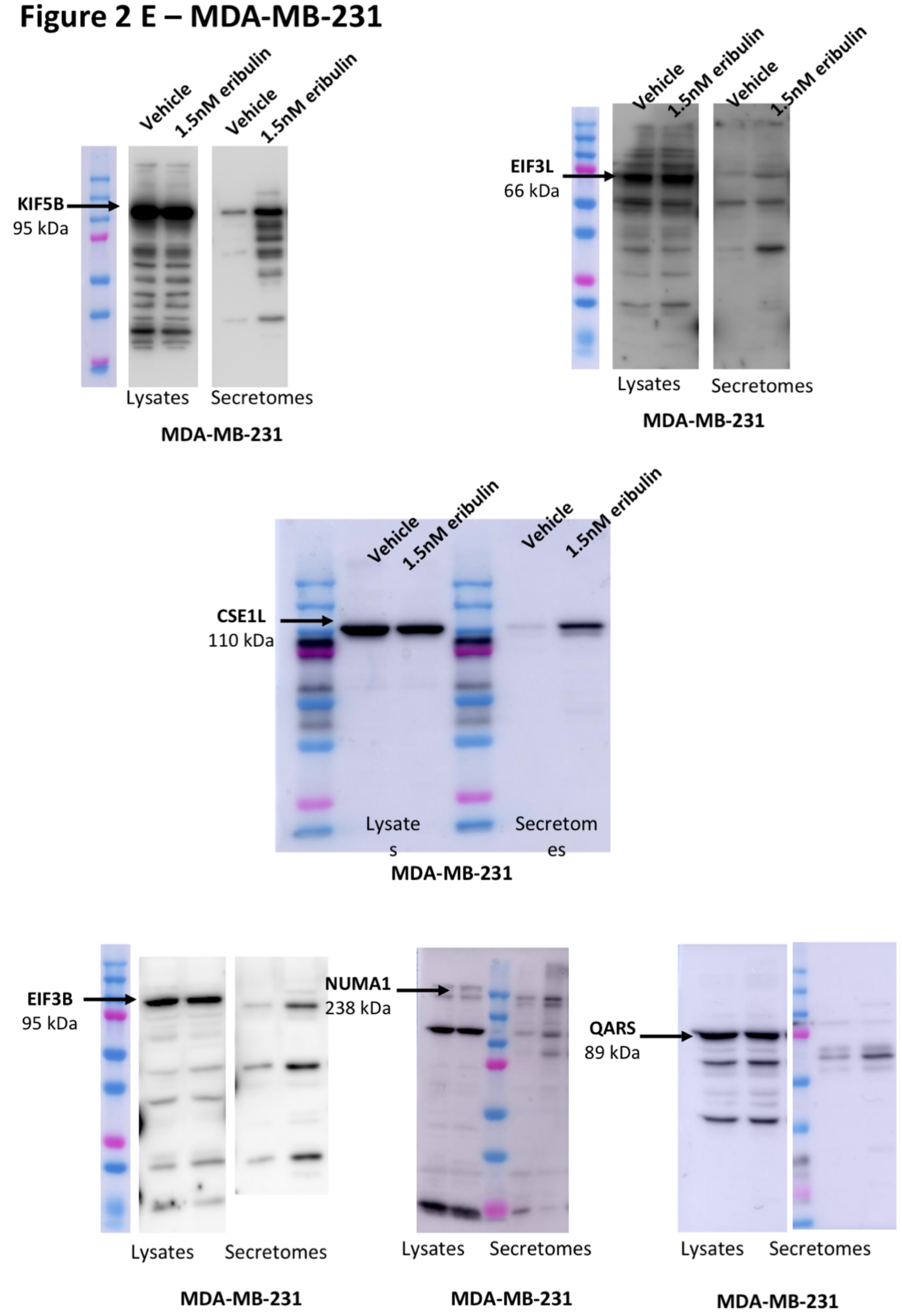

Figure 2 E – MDA-MB-231

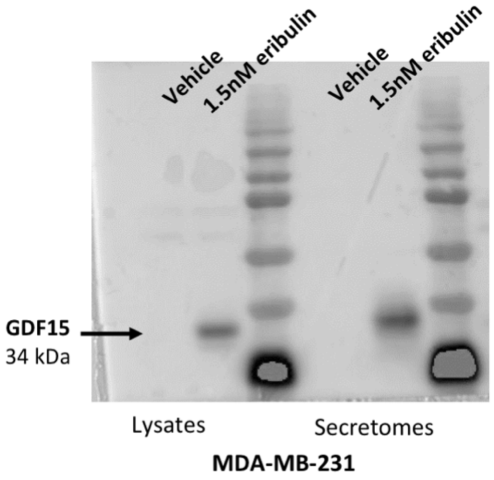

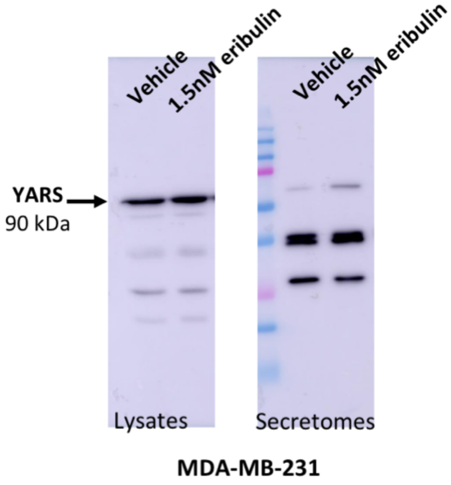

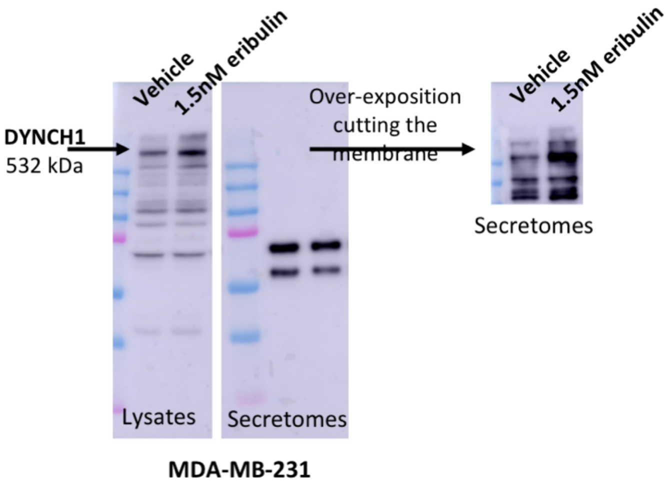

Figure 2 E – MCF7

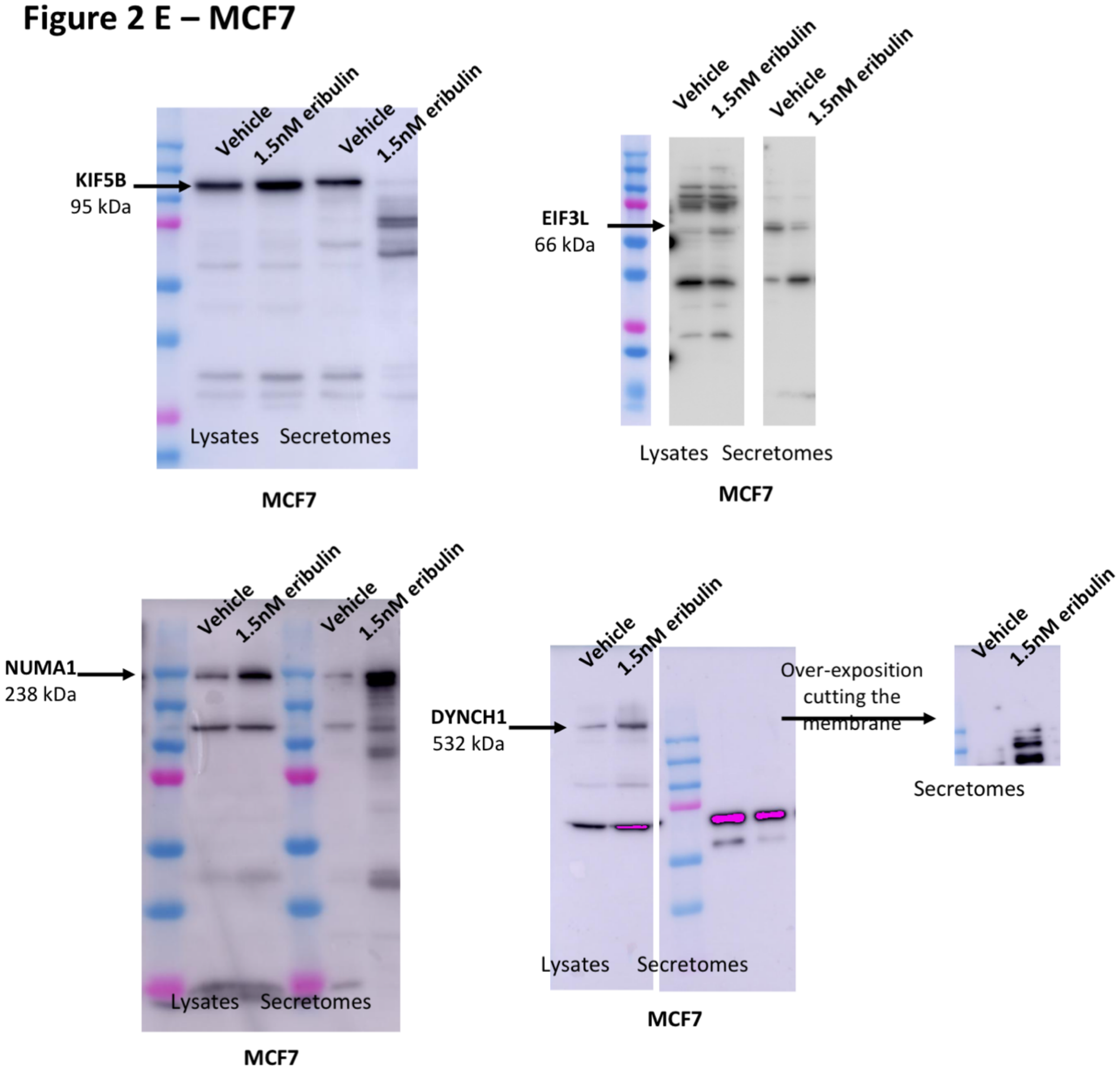

Figure 2 E – MCF7

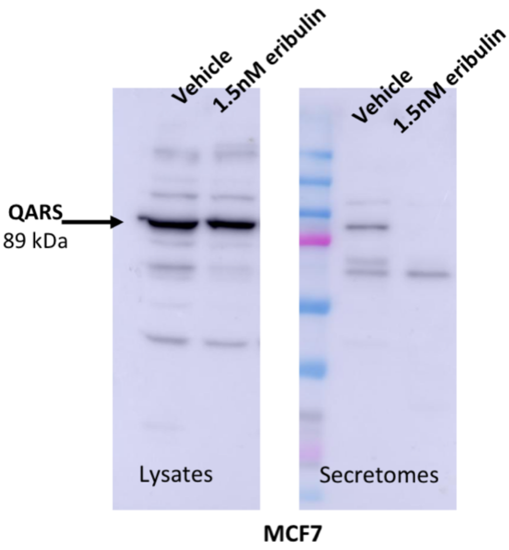

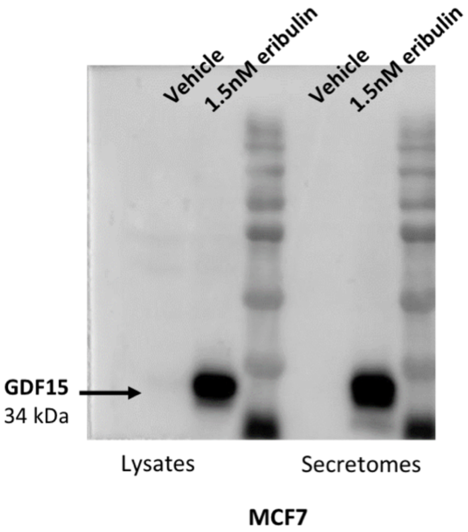

Figure 4 A

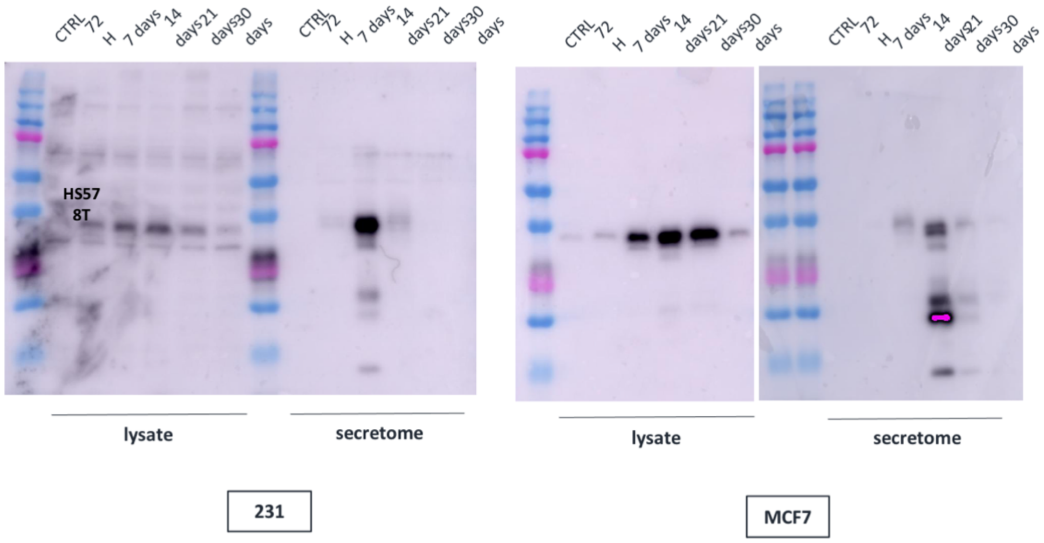

Figure 4 B

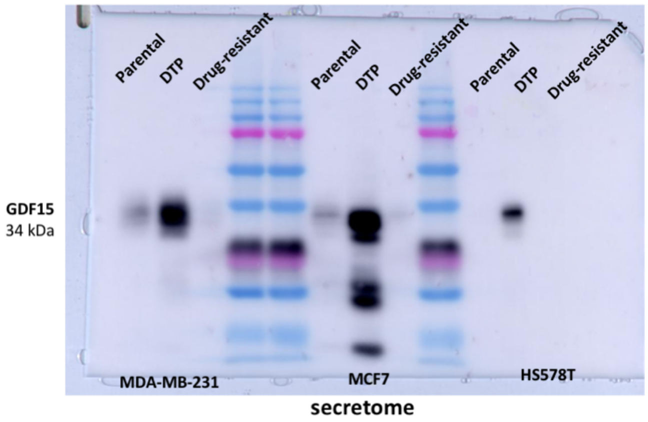

Figure 4 C

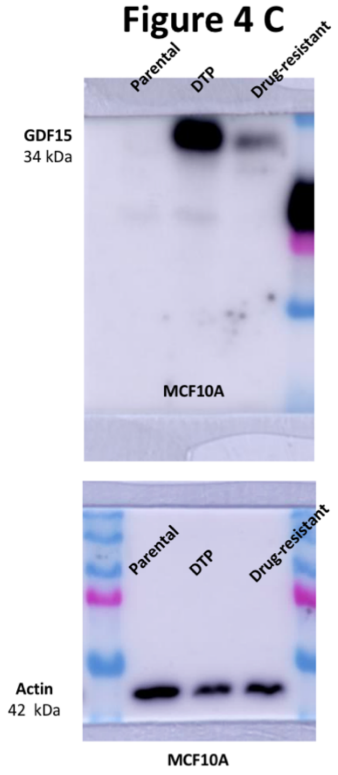

Figure 4 E

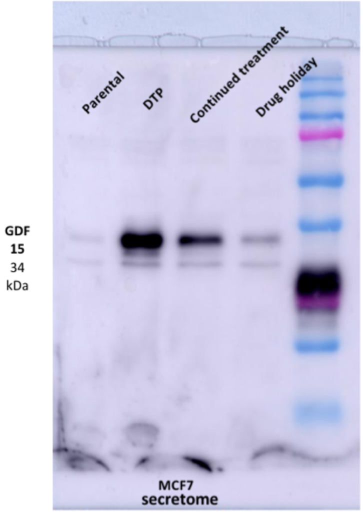

Figure 4 G

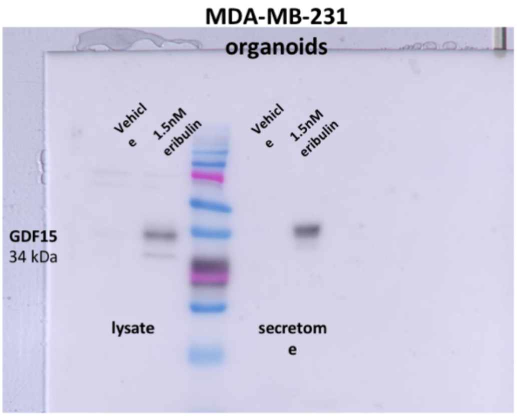

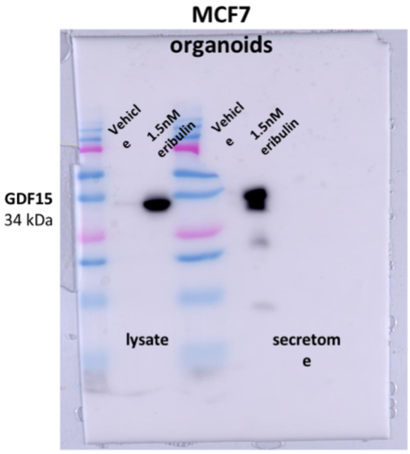

Figure 5 B

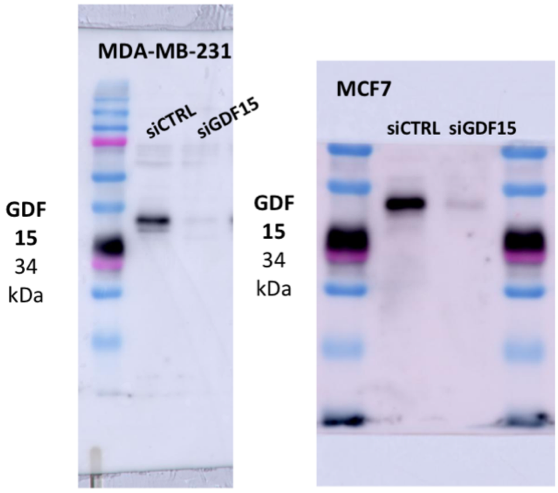

Figure 5 F

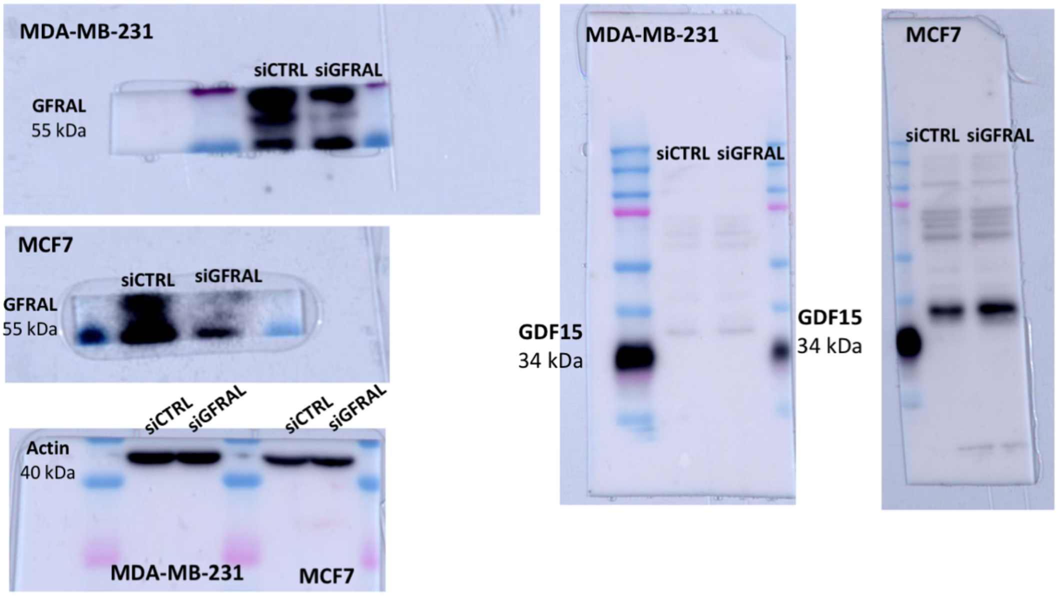

Figure S5 A

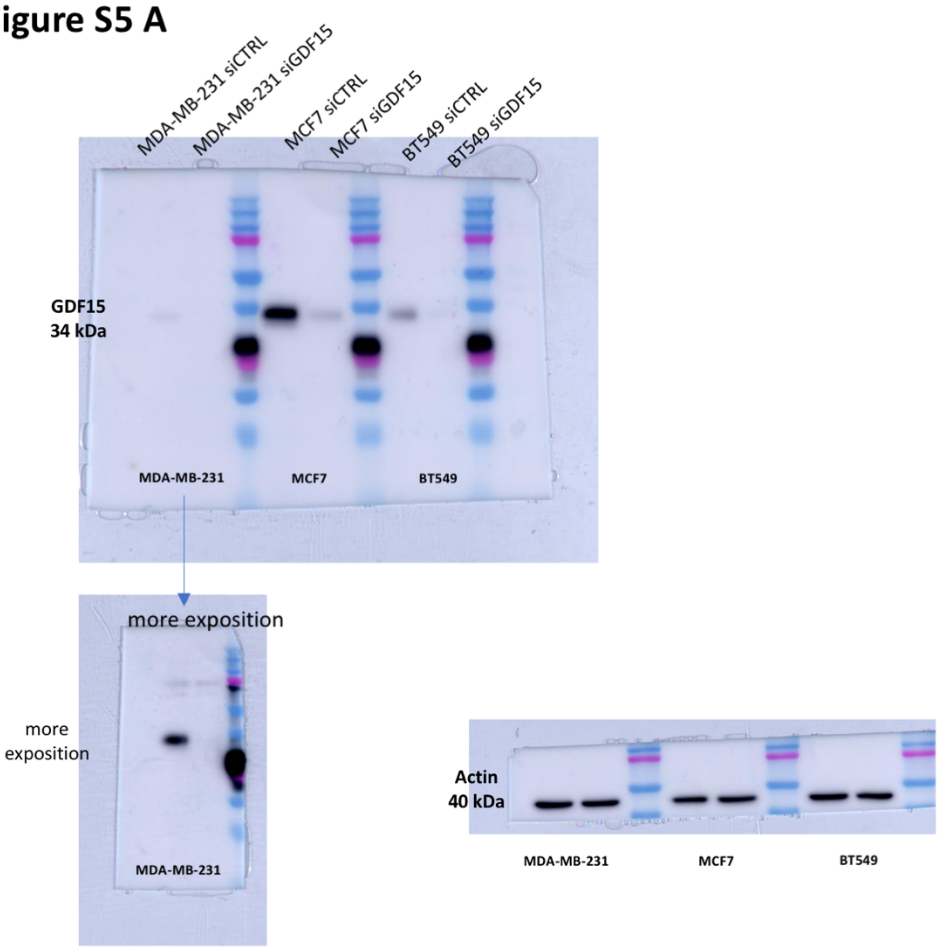

MDA-MB-231 siCTRL
MDA-MB-231 siGDF15
MCF7 siCTRL
MCF7 siGDF15
BT549 siCTRL
BT549 siGDF15
GDF15
34 kDa
MDA-MB-231
MCF7
BT549
more exposition
more exposition
MDA-MB-231
Actin
40 kDa
MDA-MB-231
MCF7
BT549

Figure S6

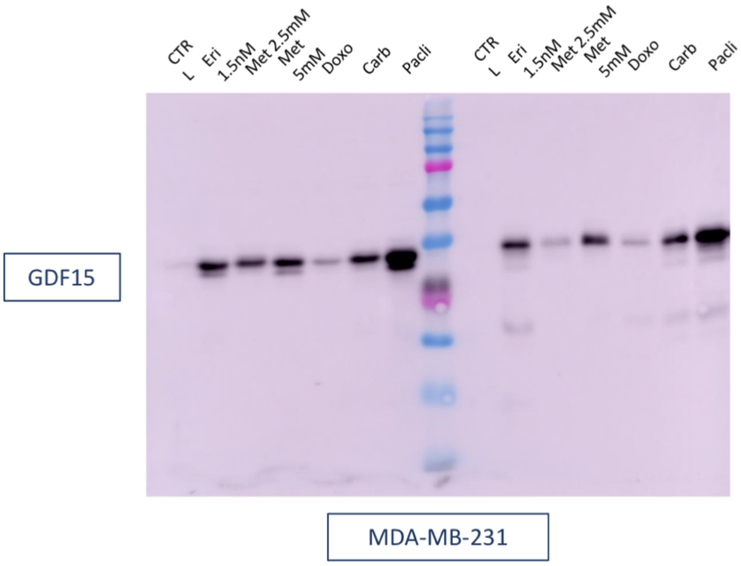
CTR
L
Eri
1.5nM
Met 2.5mM
Met
5mM
Doxo
Carb
Pacli
CTR
L
Eri
1.5nM
Met 2.5mM
Met
5mM
Doxo
Carb
Pacli
GDF15
MDA-MB-231

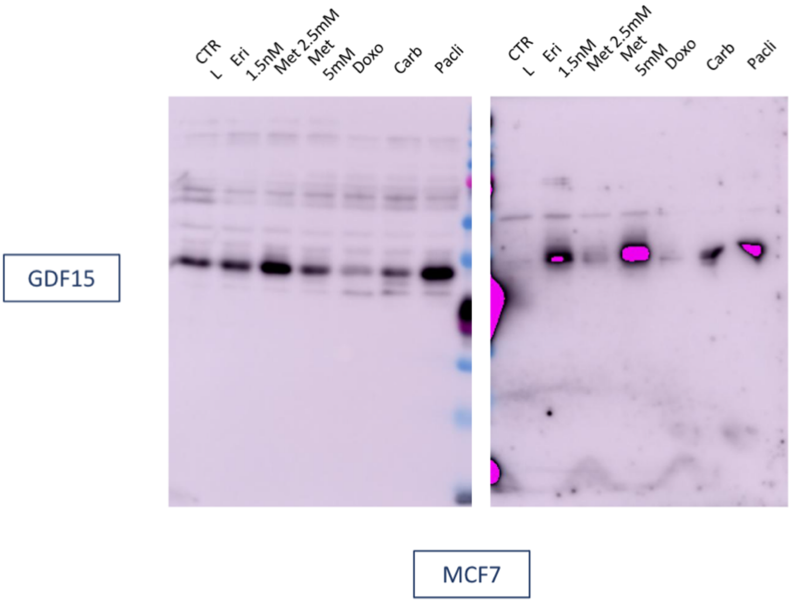
CTR
L
Eri
1.5nM
Met 2.5mM
Met
5mM
Doxo
Carb
Pacli
CTR
L
Eri
1.5nM
Met 2.5mM
Met
5mM
Doxo
Carb
Pacli
GDF15
MCF7
